# PolyUbiquitin Chain Linkage Topology Selects the Functions from the Underlying Binding Landscape

**DOI:** 10.1371/journal.pcbi.1003691

**Published:** 2014-07-03

**Authors:** Yong Wang, Chun Tang, Erkang Wang, Jin Wang

**Affiliations:** 1 State Key Laboratory of Electroanalytical Chemistry, Changchun Institute of Applied Chemistry, Chinese Academy of Sciences, Changchun, Jilin, P.R. China; 2 State Key Laboratory of Magnetic Resonance and Atomic and Molecular Physics, Wuhan Institute of Physics and Mathematics, Chinese Academy of Sciences, Wuhan, Hubei, China; 3 College of Physics, Jilin University, Changchun, Jilin, P.R. China; 4 Department of Chemistry, Physics and Applied Mathematics, State University of New York at Stony Brook, Stony Brook, New York, United States of America; University of Maryland, Baltimore, United States of America

## Abstract

Ubiquitin (Ub) can generate versatile molecular signals and lead to different celluar fates. The functional poly-valence of Ub is believed to be resulted from its ability to form distinct polymerized chains with eight linkage types. To provide a full picture of ubiquitin code, we explore the binding landscape of two free Ub monomers and also the functional landscapes of of all eight linkage types by theoretical modeling. Remarkably, we found that most of the compact structures of covalently connected dimeric Ub chains (diUbs) pre-exist on the binding landscape. These compact functional states were subsequently validated by corresponding linkage models. This leads to the proposal that the folding architecture of Ub monomer has encoded all functional states into its binding landscape, which is further selected by different topologies of polymeric Ub chains. Moreover, our results revealed that covalent linkage leads to symmetry breaking of interfacial interactions. We further propose that topological constraint not only limits the conformational space for effective switching between functional states, but also selects the local interactions for realizing the corresponding biological function. Therefore, the topological constraint provides a way for breaking the binding symmetry and reaching the functional specificity. The simulation results also provide several predictions that qualitatively and quantitatively consistent with experiments. Importantly, the K48 linkage model successfully predicted intermediate states. The resulting multi-state energy landscape was further employed to reconcile the seemingly contradictory experimental data on the conformational equilibrium of K48-diUb. Our results further suggest that hydrophobic interactions are dominant in the functional landscapes of K6-, K11-, K33- and K48 diUbs, while electrostatic interactions play a more important role in the functional landscapes of K27, K29, K63 and linear linkages.

## Author Summary

Ubiquitination, as an important post-translational modification of proteins, provides a versatile cellular signaling mechanism. This is mostly contributed by the possibility of ubiquitin units to form different polyUb chains through eight different linkages. However, it is still unclear how these linkage types determine the different functions of polyUb chains. In this study, we address this question via the theoretical modeling and molecular dynamics simulation. This allows us to obtain a full picture of topology-function relationship of polyUb chains. The theoretical results led us to propose that topology of polyUb chains selects the functional landscapes from its binding landscape and the topological constraint provides a way for breaking the binding symmetry and reaching the functional specificity.

## Introduction

Ubiquitin (Ub) was discovered in the mid-1970s [1] and has been found to ubiquitously exist in eukaryotes. Ub plays a central role in regulating the balance between a protein's destruction and its synthesis. The dysfunction of Ub is closely linked to a wide range of disorder diseases (including Alzheimer's, Parkinson and Prion diseases and others) [2]. Besides the well-known function of protein degradation, Ubs also serve as numerous regulatory signals including endocytosis, DNA repair, autophagy and transcription [3]. Most signal functions of Ub can be understood by considering it as a “molecular tag” which marks a protein and determines the fate of this post-translationally modified protein.

Ubiquitin tag is achieved via covalent attachment to a substrate protein with a monomeric Ub (monoubiquitination), multiple Ubs (multi-monoubiquitination) or a Ub polymer (polyubiquitination) [4]. In a poly-Ub chain, Ub units are assembled with each other through forming covalent bonds between the carboxyl-terminal group of one Ub (termed the distal moiety) and the side-chain 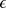-amino group of a lysine among the all seven lysines (K6, K11, K27, K29, K33, K48 and K63) or the amino-terminal residue (M1, corresponding chains often referred to as linear) of another Ub (termed the proximal moiety). It is well established that all ubiquitin linkage types coexist in all cells with varying abundance [5]–[7]. Remarkably, almost half are populated by K48 and K63 linkage types whose cellular functions have been well characterized [8]. Extensive studies suggested that the former usually takes action in proteasomal degradation (the most common fate of a ubiquitinated protein), while the latter plays non-degradative roles in cell signalling, such as endocytosis and DNA damage repair [8]–[10]. Beside the two typical linkages, K11 linkage is also abundantly present in cells. A few recent work reported that K11 linkage chain not only has non-degradative roles but also acts as potent proteasomal degradation signals in diverse cellular pathways [11]–[13]. This is a surprise finding because K48-linked chains have always been considered to be the unique destruction tag for unneeded proteins in cells. By contrast, very little is known about the remaining five atypical linkage types [4], [14], [15].

**Figure 1:**
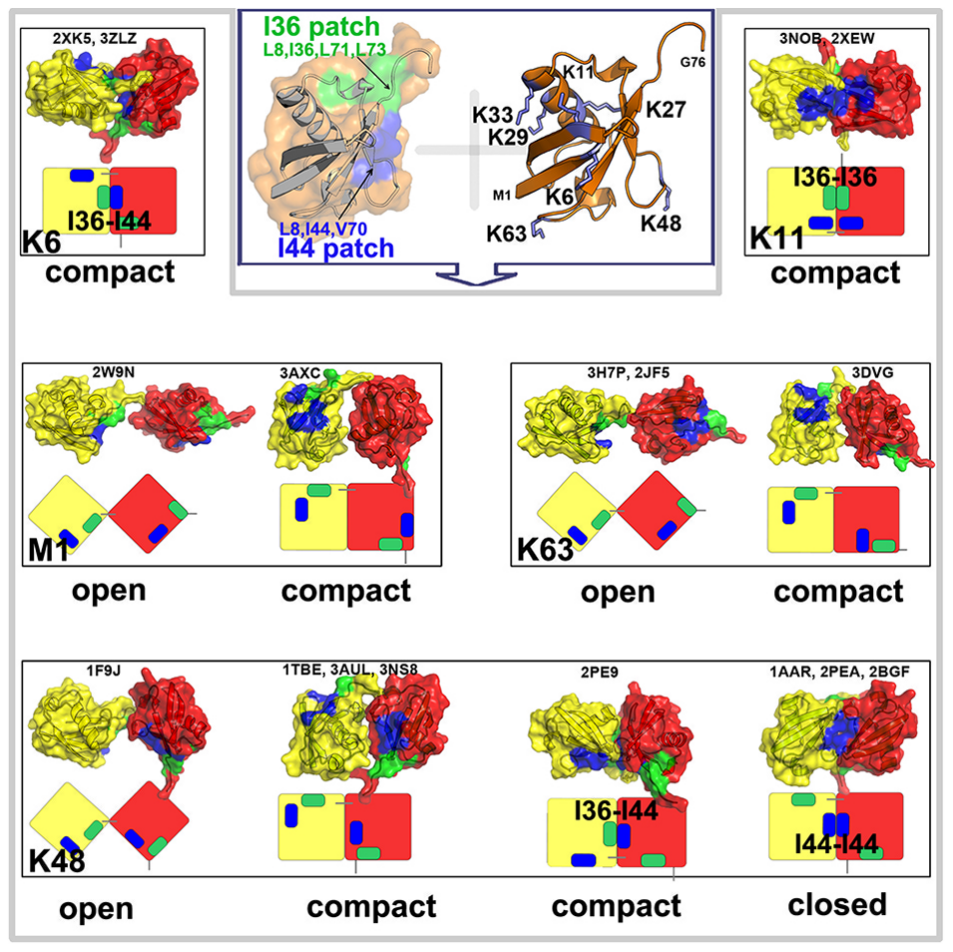
Experimental structures of diUbs with different linkages. Only five linkage types have been structurally characterized (summarized in Table 1 in Text S1). The corresponding structures are shown with the distal Ub unit (contributes a carboxyl group of G76 to form the linkage) in yellow and the proximal Ub unit (contributes an 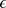-amino group of lysine) in red, above a schematic cartoon. The formation of Ub interfaces is mainly contributed by two hydrophobic patches. One is the I44 patch (color in blue) consisting of L8, I44, V70, another is the I36 patch (colored in green) involving L8, I36, L71 and L73. These experimental structures include: compact structure of K6-linked diUb (2XK5, 3ZLZ), compact structure of K11-linked diUb (3NOB, 2XEW), open and compact structures of M1-linked diUb (2W9N, 3AXC, respectively), open and compact structures of K63-linked diUb (2JF5 and 3H7P, 3DVG, respectively), and four distinct structures of K48-diUb consisting of open (1F9J), closed (1AAR) and two compact conformations (1TBE, 3AUL, 3NS8 and 2PE9, respectively).

At present, it seems clear that different polyUb chains generate distinct molecular signals and lead to different cell fates [16]. But how do these linkage types determine the different functions of Ub chains and the diversity of ubiquitin recognition? The answer to this question seems to lie in the structure of Ub chains and their fluctuations (conformational dynamics) by considering the fact that all types of polyUb chains are constructed by identical Ub units with the same physicochemical properties (mass, charge and interactions) but different topology (linkage position and length). In fact, the topology of an Ub polymer has been suggested to be important in the control of the fate of a Ub modified protein by a few experimental and theoretical studies [17]–[19]. However, it is still unclear how the topology affects the underlying energy landscape of polyUb chains themselves. Great efforts have been made in the elucidation of conformational diversity between alternatively linked polyUB chains. In summary, there are five linkage types (including M1, K6, K11, K48 and K63, see Fig. 1, as well as Table 1 in Text S1) which have been structurally characterized on the basis of traditional biophysical tools, such as X-ray crystallography, nuclear magnetic resonance (NMR) [20] and small angle X-ray scattering (SAXS) [21]. In addition, a most recent work carried out by Tang and coworkers reported the conformational dynamics of free Ub monomers in solution by using paramagnetic relaxation enhancement (PRE), an NMR techniques sensitive to lowly populated species [22]. This PRE study has suggested that Ubs can form non-covalent dimers with a modest binding affinity [22]. This reveals that Ubs not only interact with ubiqtitin-binding domains in the cell, but also are able to interact with themselves to form dimeric molecules, whose role is nonnegligible in the case of high concentration. All these data provided strong evidence that the conformational behaviours of Ub monomers and polymers are a lot more complicated than originally thought.

Structural characterizations can provide important local information (corresponding to energy minima or metastable states) on the functional landscape at the bottom of “energy funnel” [23], [24]. For a deeper understanding that how Ub system functions, however, it is essential to obtain a global picture through the functional landscape [24]. This presents a unique chance for theoretical modelling and simulations. It is well-known that a protein in nature is marginally stable through the balance of interactions (folding and binding interactions). This is especially true for a protein complex or a multi-domain protein which functions via frequent binding and unbinding events between folded units. These events are largely driven by two types of interactions, that is, electrostatic and hydrophobic interactions [25]. For polyUb systems, this notion is also strongly supported by two facts. On the one hand, at near-physiological conditions, Ubs can form compact interfaces involving numerous hydrophobic residues [22], [26], highlighting the important role of hydrophobic interactions in the Ub assemble. On the other hand, the conformational dynamics of polyUb can be highly dependent on the environmental pH [17], [20], [27], indicating the importance of electrostatic interactions. However, it is still unknown about their relative contributions in the association of Ub units and their relationships to the distinct functional landscapes of Ub chains.

In the present work, we will develop a flexible binding model by the introduction of electrostatic and hydrophobic interactions and employ it to explore the functional landscape of polyUb chains. Note that only two Ub units (diUb) were used in our model because this is the simplest form of a polyUb chain and the minimal structural unit for longer polyUb chains. Different polyUb chains with all seven lysine linkages and linear linkage as well as free Ub monomers (without a linkage) were investigated based on the flexible binding model. This model allows us to determine the dominant driving forces in the assembly of diUbs with different linkages. The simulation results provide several predictions that qualitatively and quantitatively consistent with experiments. Importantly, the functional landscape of K48-diUb is predicted to have three intermediate states. Inspired by the multi-state functional landscape, we employed a simple three-state model to well reconcile the seemingly contradictory experimental data on the conformational equilibrium of K48-diUb.

## Results

The electrostatic interactions in our model were calculated by the Debye-Huckel model whose parameters have been carefully tested in our previous works [24], [28]. To further model the hydrophobic interactions (see **Methods**), we then introduced 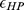 to account for the strength of hydrophobic forces which was calibrated according to available experimental data, especially the apparent binding affinity between Ub units which has been measured to be about 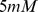 [22]. Based on this parameter sets, we performed molecular dynamics simulations to explore the conformational dynamics of polyUb chains.

### Compact conformations of covalently bonded diUbs pre-exist on the binding landscape of free ub monomers

First, we investigated the conformational dynamics of two free Ub monomers by performing MD simulation based on the free Ub model. Hereby, we point out that in the free Ub model, the two Ub monomers are not connected by a covalent bond. This free model simulation is important not only because our work is the first to simulate the dimerization of free Ub monomers, but also because the results will provide us a benchmark to estimate and quantify the effects of linkages on the conformational behaviour of diUb chains. After calibrating the energetic parameters in the free model, we then constructed the corresponding covalent linkage models to investigate the conformational dynamics of diUbs with all linkage types. These linkage models were carried out by introducing an isopeptide or peptide bond between G76 of one Ub monomer and one of its seven lysine residues (by 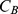 bead) or the N-terminal M1 (by 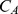 bead) of another Ub monomers on the basis of the free model (see Table 2 in Text S1 and **Methods**),

To assess the conformational space sampled by our flexible binding model, we plotted the free energy surfaces as a function of the distance between the center of mass of Ub monomers (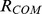) and the RMSDs from available structures resolved by X-ray crystallography and NMR (see Fig. S1). The results show that most of the experimental structures can be sampled by the free model and further validated by the corresponding linkage models. We further calculated the minimal RMSD of Ub dimers from all experimental structures (see Table 3 and 4 in Text S1). It shows that all these structures have minimal RMSD less than 0.35 nm in the free model and less than 0.25 nm in the corresponding linkage models. It is unexpected to us given the fact that the huge conformational space of two free Ub monomers was explored with limited computational time, despite that most of them are not located at the free energy basins in the free model.

**Figure 2:**
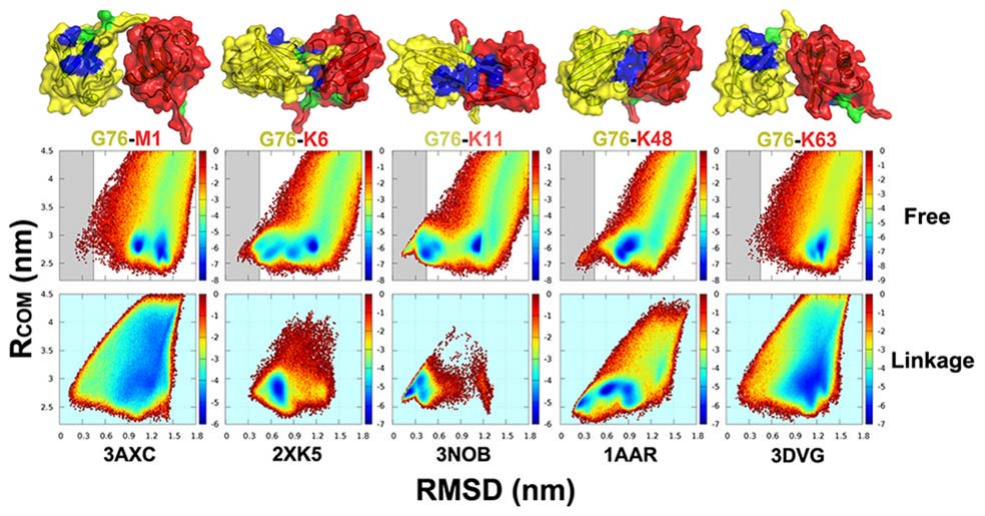
Compact conformations of diUbs preexist on the binding landscape of free Ubs. The free energy surfaces as a function of the distance between the center of mass of Ub monomers (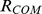) and RMSDs from the compact structures (PDB 3AXC, 2XK5, 3NOB, 1AAR and 3DVG) of five linkage types resolved by X-ray crystallography and NMR (M1, K6, K11, K48 and K63, listed in Table 1 in Text S1). The native conformational regions are labelled by grey in the free Ub model. Note that the same conformational space sampled by the free Ub model at given concentrations (5 mM here) was used. For comparison, the results of corresponding linkage models (CGM1, CGK6, CGK11, CGK48 and CGK63 models, see Table 2 in Text S1) are also plotted below. The compact structures of these linkage types are shown above with the distal Ub unit in yellow and the proximal Ub unit in red. The two hydrophobic patches, I36 and I44, are colored in green and blue, respectively.

Furthermore, the results show that the compact structures in contrast to open structures were better captured by the corresponding linkage models. Remarkably, the good characterization of compact structures was also able to be achieved by the free model with a given protein concentration (5 mM in the present work). To emphasize this point, free energy surfaces projected onto 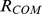 and RMSD from compact structures are shown in Fig. 2. It indicates that the compact structures of M1-, K6-, K11-, K48- and K63-linked diUbs have remarkable populations on the conformational space. Especially for K6, K11, and K48 linkage types, we found that there are free energy basins located at or near the native conformational region (typically with RMSD from the compact structures less than 0.4 nm) on the binding landscape sampled by the free model and the functional landscape sampled by their linkage models. As a control, we projected the conformational space sampled by the free Ub model onto RMSD from a dimeric structure only stablized by crystal packing forces (so represents a “wrong” structure), as shown in Fig. S2. It shows that the free Ub monomers never sample such conformation. It therefore supports that the assembly of free Ub monomers to the conformations similar to the experimental structures of diUbs is far beyond an accidental event. This leads to a remarkable finding that Ub monomers without covalent linkages have the ability to assemble the native structures of polyUb chains, and these assembled conformations are further stabilized by the formation of linkages between Ub units.

### Linkage topology constraint leads to intefacial symmetry breaking

**Figure 3:**
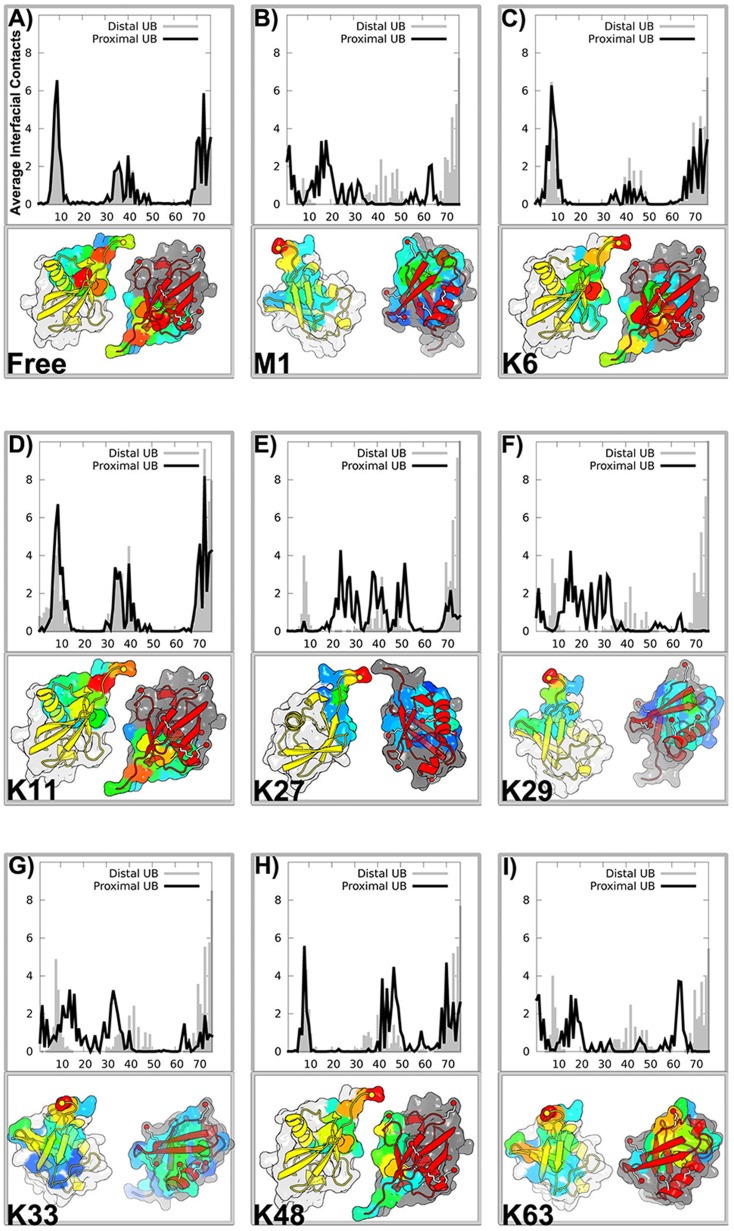
Symmetry of interfacial interactions present in the free model is broken in the linkage models. It shows the distribution of average interfacial contacts along the residue index of proximal Ub (black) and distal Ub (grey). The distribution was mapped onto the surface of two Ub monomers whose positions are artificial for better view of the binding surface. The distal unit is represented by light grey surface with yellow cartoon, while the proximal unit by dark grey surface with red cartoon. The “hot spot” residues taking part in the binding are highlighted by surface with colors from blue to red, corresponding to having low and high interfacial contacts, respectively. Note that the analysis was based on the conformations with 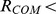
3.2 nm.

To further shed light on molecular or microscopic details of the assembly process of Ub units, we measured the interfacial interactions by counting the inter-molecular contacts. Fig. 3A shows that the two Ub molecules form noncovalent dimeric conformations through a wide interface composed of residues K6-K11, E34-P37, Q40, R42, I44, G47, H68-G76. This result is in good agreement with the PRE experimental data [22] which indicates a symmetric interface encompassing residues 4–12, 42–51 and 62–71 with the exception of E34-P37 region and the C-terminal tails. This could be a result of insufficient number of paramagnetic tags used for PRE measurement [29]. In fact, the later two regions contain several important hydrophobic residues including I36 and L73 which are members of another hydrophobic patch, referred to as the I36 patch [4]. In other words, our simulation results highlight the important role of two hydrophobic patches in the formation of Ub interfaces. One is the well-known I44 patch, and another is the I36 patch involving L8, I36, L71 and L73. In fact, the I36 patch has been found to be sequestered in the interface of K6- and K11-linked polyUb chains as shown in three X-ray structures (PDB 2XK5, 3NOB and 2XEW, see Fig. 1).

Fig. 3A also indicates that the interfacial contact distributions of two Ub monomers are perfectly overlapped. This is expected because our model does not introduce any biasing to a particular assembled structure. The identical distribution of interfacial interactions not only reflects the sufficient sampling of the simulations, but also indicates the symmetry of interfacial interactions between Ub units. The symmetry is also supported by the interfacial contact matrix, as shown in Fig. S3. To monitor the effect of covalent linkages on the binding of two Ub units, we further inspect the interfacial contact distributions of diUbs with all linkage types, as shown in Fig. 3B–I. By comparison with the data obtained by the free model, it clearly indicates that the symmetry of interfacial interactions present in the binding of free Ub monomers is broken by the introduction of a covalent bond between two Ub units.

We may ask what is the physical reason (entropic or enthalpic) of the symmetry breaking? Or whether the symmetry breaking has its biological benefits? In contrast to the free model, the only difference lies in an extra isopeptide/peptide bond introduced between Ub units in each linkage model. The free energy contribution of the bonded constraint was further quantified on the basis of polymer theory [30] (see Fig. S4). The result suggested that the impact is mainly entropic rather than enthalpic. In fact, in contrast to the binding free energy landscape of two Ub monomers, the functional landscapes of covalently linked diUbs are significantly more compact (with a smaller average 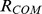).

Furthermore, to quantify the relative entropic and enthalpic contributions of the bonded constraint to the functional landscapes of different linkage types, we performed a detailed analysis of entropy-enthalpy compensation and calculated the correlation coefficients between entropy and free energy (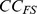), as well as the correlation coefficients between enthalpy and free energy (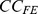). The results are shown in Fig. S5, S6, S7 and Table 5 in Text S1. By comparison with the free model, 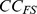 increases in all linkage models, but 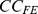 is dependent on the linkage types. Specially, for M1, K27, K29 and K63 linkage types, the bonded constraint reduces 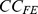 and increases 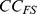. Therefore, their interfacial symmetries are broken by entropy. For K6 and K11 linkage models, the degree of the increase of 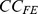 is significantly larger than the degree of the increase of 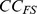. So their interfacial symmetries are mainly broken by enthalpy. While for K33 and K48 linkage models, neither 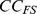 nor 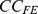 shows strong correlations, but the degree of 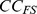 increase is much larger than the degree of 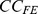 increase. Thus this case was considered to be entropically driven.

In summary, the quantification analysis of entropy-enthalpy compensation supports the proposal that the symmetry breaking arises mainly from entropy rather than enthalpy for most linkage types. Entropy reduction breaks the symmetry of interfacial interactions by decreasing the degrees of freedom of Ub system so as to facilitate the searching of functional states on the functional landscape of polyUb chains.

### Relationships between interfacial hydrophobic/electrostatic interactions and conformational distribution

By inspecting the compact or closed structures of diUbs, we found that charged residues are also involved in the formation of interfaces in addition to hydrophobic residues. For example, in the closed state of K48-diUb (PDB 1AAR), the hydrophobic interface formed between I44 patches contains three basic residues R42, K48 and H68. More importantly, there are not any negatively charged residues located at the opposite face of these positively charged residues. This indicates that electrostatic interactions may play a negative role in the formation of hydrophobic interface between I44 patches. However, this does not rule out the possibility that electrostatic interactions contribute to form interfaces at other regions of the Ub surface. Now there are fundamental questions: which interactions are dominant for Ub-Ub binding? And what are their roles in diverse functional landscapes of diUbs?

To investigate the relative contribution of hydrophobic and electrostatic interactions and their relationships with the interface formation in the binding of Ub units, we decomposed the interfacial energy of the system into two terms, that is, hydrophobic energy 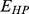 and electrostatic energy 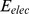. Then we examined their respective distributions and correlations.

Fig. S8A and B show the free energy profiles as a function of 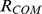 and 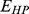 and of 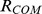 and 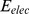. By comparison of the free energy profiles, it indicates a physical picture as expected, that electrostatic force is long-ranged, while hydrophobic force is short-ranged. We further show the free energy profiles as a function of 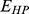 and 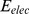 in Fig. S8C. It indicates that 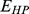 is negatively related to 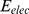, implying a competition between hydrophobic interactions and electrostatic interactions in the formation of Ub-Ub bound complex. This picture becomes clearer when investigating the distribution of 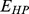 and 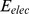 as a function of the distance between I44 hydrophobic patches of two Ub monomers (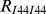), as shown in Fig. S8D. The energy distribution clearly shows that the I44-I44 interface is highly favored by hydrophobic interactions, but it is disfavored by electrostatic interactions. This is consistent with the structural analysis, indicating the interface is surrounded by three basic residues (R42, K48 and H68), whereas no acid residues counterbalance to the net charges.

**Figure 4:**
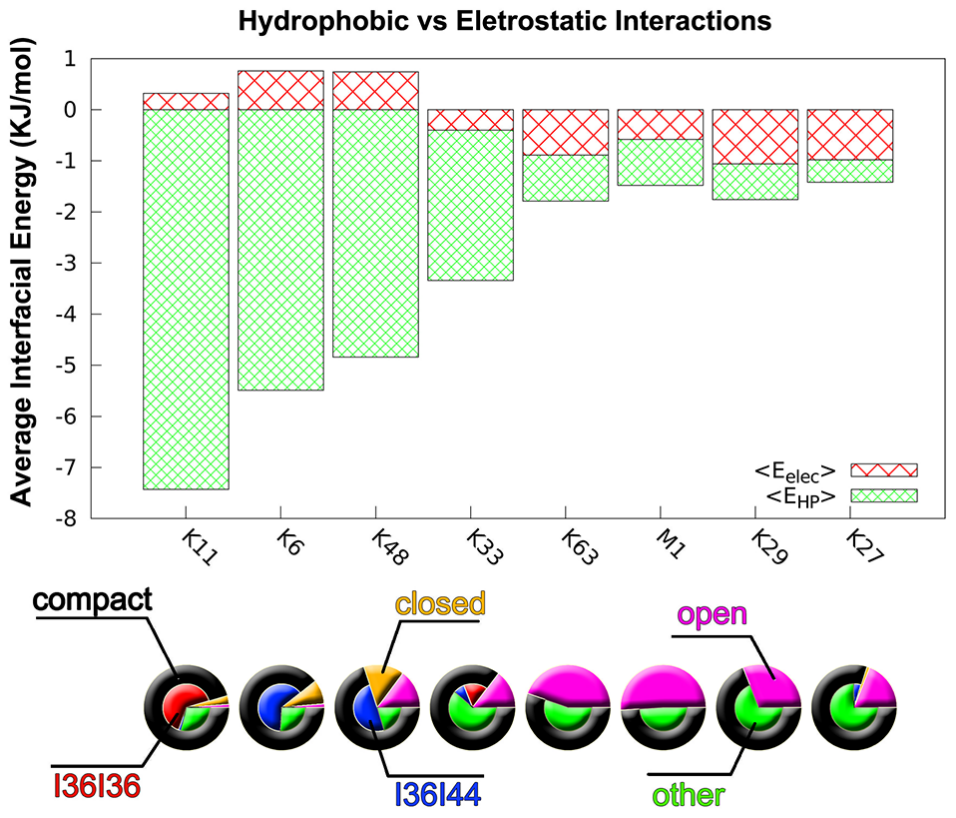
Relationship between conformational populations and interfacial interactions (hydrophobic and electrostatic interactions). The interfacial potential energy is decomposed into hydrophobic energy 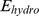 and electrostatic energy 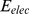. The energy distribution of average 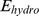 and 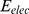 of all eight linkage types is shown. The population distribution of open, closed, compact states is represented by magenta, orange and black pies, respectively. The compact state (black) is further decomposed into the I36I36 (red), I36I44 (blue) and other states (green). See quantitative results in Table 1.

Fig. 4 shows the relationship between conformational populations and interfacial interaction. We can see that hydrophobic interactions play a more dominant role in K11-, K6-, K48- and K33-linked diUbs than other linkage types. Especially for K11, K6 and K48 linkages, electrostatic interactions made a negative contribution (positive energy) to formation of compact diUbs. It also indicates the competition between electrostatic interactions and hydrophobic interactions in the formation of compact structures. This finding is also consistent with the results from free Ub model which suggests that 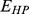 is negatively related to 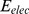 as shown in Fig. S8. In fact, around the I36 and I44 hydrophobic patches there are several basic residues which may form electrostatically repulsive force as the hydrophobic patches are buried at the interface. Given the strong pH dependence of conformational equilibrium of K48 linkage evident from solution experiments [20], [26], [27], we expect that the conformational distribution of K6 and K11 linkages highly dependent on pH as well. We hypothesize that, decreasing pH will open their conformations which may be validated by further experiments in the future. By contrast, electrostatic interactions are more important to the association of Ub units in K27, K29 and K63 as well M1 linkages.

To quantify the differences between the conformational space of diUbs with all types of linkages, we calculated the population of conformational states based on a three-state model derived from the free energy landscape of K48-diUb (details shown in following subsection). The conformational space was coarse-grained into three states: open, closed and compact. The compact state can be further divided into I36-I36, I36–I44 and other compact state according to the role of I36 and I44 hydrophobic patches in the formation of interface between Ub units. Note that the criteria for determining the open, closed and compact states are defined according to the corresponding free energy profiles (Fig. S9). The results are summarized in Table 1 (and also Fig. 4). It supports the open populations in this order: first M1 (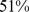), K63 (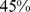) and K29 (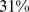), then K27 (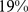), K48 (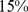) and K33 (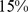), and finally K6 (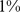) and K11 (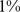). If according to the closed populations, Ub chains can be categorized into two groups. For the first group the order is first K48 (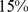 closed), subsequently K6 (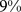), then K11 (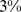) and finally K27 (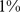). For the second group including M1, K29, K33 and K63, they are not able to form closed conformation. This finding is fully consistent with the previous studies [31].

**Table 1:** Relationships between electrostatic/hydrophobic interactions and conformational distribution of diUb chains.

Model	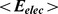	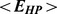	Open^a^	closed^b^	Compact^c^	= (I36I36^d^+I36I44^e^+other)
CGK6	0.76 (0.02)^f^	−5.49 (0.06)	1.4 (0.3)	9.3 (0.4)	89.3 (0.3)	0.01 (0.03)+63.3 (1.3)+26.0 (1.3)
CGK11	0.32 (0.01)	−7.47 (0.05)	1.1 (0.2)	3.4 (0.2)	95.6 (0.3)	65.6 (1.0)+1.3 (0.3)+28.7 (1.1)
CGK27	−1.01 (0.02)	−0.37 (0.10)	19.4 (0.7)	1.0 (0.2)	79.6 (0.5)	0.1 (0.05)+4.4 (0.7)+75.2 (0.6)
CGK29	−1.06 (0.01)	−0.70 (0.04)	31.2 (0.6)	0.0	68.8 (0.6)	0.0+0.0+68.8 (0.6)
CGK33	−0.40 (0.04)	−2.95 (0.17)	14.4 (1.1)	0.0	85.6 (1.1)	16.2 (2.9)+8.1 (0.9)+61.4 (2.5)
CGK48	0.74 (0.02)	−4.84 (0.05)	15.0 (0.8)	15.1 (1.2)	69.9 (1.6)	0.0+49.7 (2.0)+20.2 (0.7)
CGK63	−0.89 (0.02)	−0.90 (0.02)	44.8 (1.0)	0.0	55.2 (1.0)	0.0+0.0+55.2 (1.0)
CGM1	−0.58 (0.01)	−0.90 (0.02)	50.9 (0.6)	0.0	49.1 (0.6)	0.0+0.0+49.1 (0.6)

a Open state is defined as the conformations with 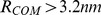.

b Closed state is defined as the conformations with 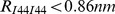.

c The remaining conformational space with the exception of open and closed states is defined as compact state. The compact state can be further divided into I36-I36, I36–I44 and other compact state according to the role of I36 and I44 hydrophobic patches in the formation of interface between Ub units.

d The I36-I36 state is considered to be formed as 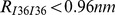 and 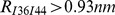 as well as 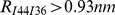.

e The I36–I44 state is considered to be formed as 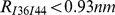 or 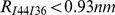, and 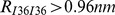 as well as 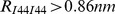.

f Standard deviations (SDs) are given in parentheses. SDs were measured from eight long independent simulations with the same parameter sets.

In addition, we predicted that K33-diUb is able to form I36 patches involved hydrophobic interface like K11-diUb. More precisely, the population of I36-I36 compact state for K33 is 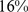 less than that of K11 of 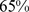. While K27 and K48 linked diUbs in addition to K11 and K33 linked diUbs have the ability to form hydrophobic interfaces between I36 patch and I44 patch. Their populations of I36–I44 compact state are in this order: K6 (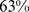), K48 (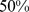), K33 (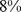), K27 (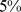) and K11 (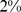).

By integrating the entropy-enthalpy compensation analysis, we found that, the long-range electrostatic interactions play a remarkable role in entropy-driven cases (such as K63, M1, K29 and K27) with significant population of open state, while the short-range hydrophobic interactions dominate in enthalpy-driven cases whose conformational spaces are tend to be compact. This implies an inherent relationship between entropy/enthalpy, electrostatic/hydrophobic interactions, and conformational distributions.

### Predicted multi-state functional landscape of K48-diUb can reconcile the seemingly contradictory experimental data

Among the eight different polyUb chains, K48-linkage is the best characterized. K48-linked diUb has been suggested to have multiple distinguished structures by X-ray crystallography and NMR (see Fig. 1). Previous and recent NMR spectroscopy experiments have collected abundant data (including chemical shift perturbation, residual dipolar coupling, and relaxation) on conformational behaviour of K48-diUb in solution [20], [26], [27], [32]. These data provide strong evidence that K48-diUb cannot be described by a single conformational state, instead its I44-involved hydrophobic interface rapidly opens and closes on 10–40 ns time scale [33]–[35], implying multiple possible free energy basins at the functional landscape.

Previous experimental data also suggested the high pH-dependence of the conformational equilibrium [20], [26], [27]. Despite the fact that lowering pH will increase the population of open state [26], it is still a matter of debate whether the open state is predominant at physiological conditions. For example, Varadan et al. found the population of open state is 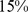 [26], but Hirano et al. concluded to be 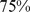 [20]. The present work will try to reconcile the seemingly conflicting observations by investigating the conformational dynamics of K48-diUb with CGK48 model (two Ub monomers linked by a K48-G76 isopeptide bond, see Table 2 in Text S1).

**Figure 5:**
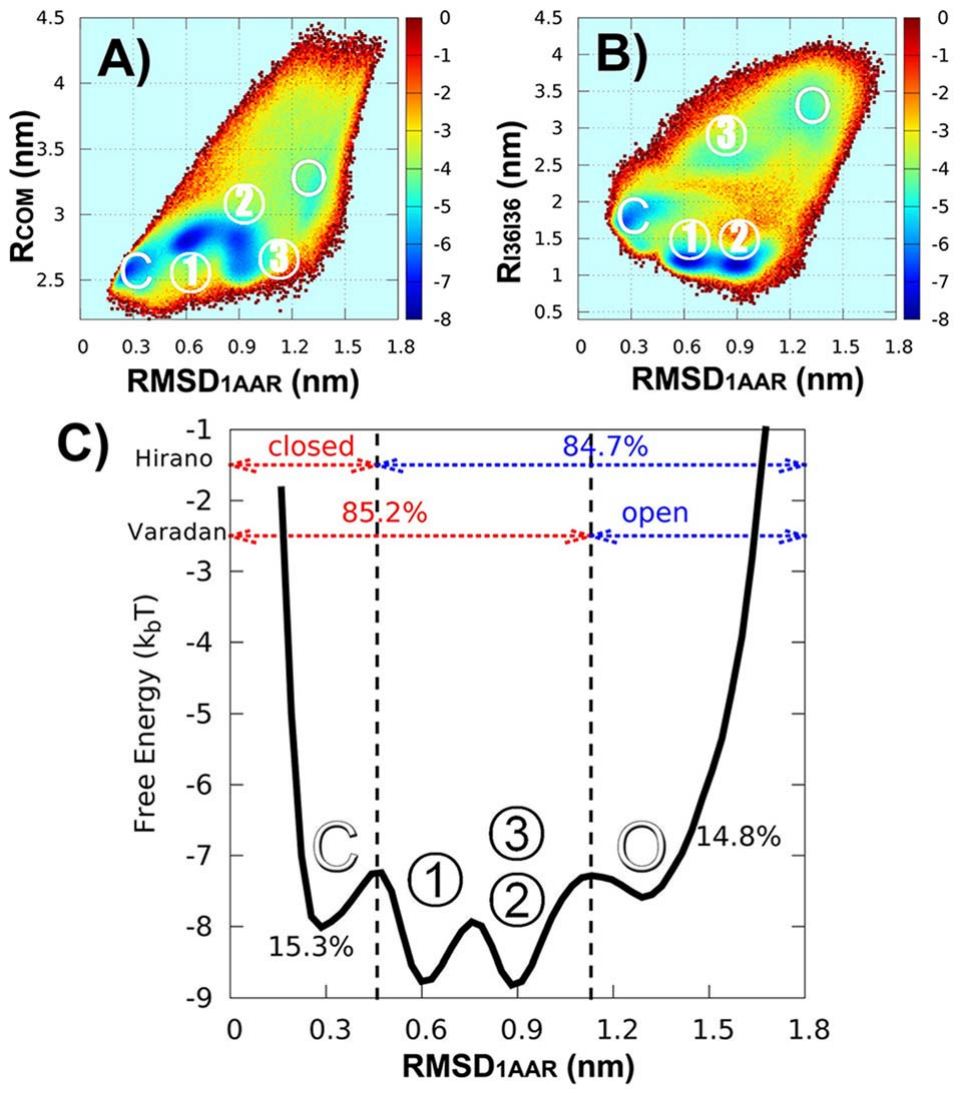
Multi-basin functional landscape of K48-diUb can be used to well reconcile the seemingly contradictory experimental measurements of the conformational equilibrium. (A) Free energy profile as a function of 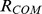 and RMSD from the X-ray structure of the closed form of diUb (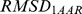). (B) Free energy profile as a function of the distance between I36 hydrophobic patches (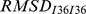) and 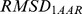. Beside the closed and open basins (labeled by O and C), there are three intermediate basins (labeled by 1, 2, 3) on the free energy surface of K48-diUb. (C) Free energy profile as a function of 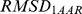. Intermediate states 2 and 3 are indistinguishable in the one-dimensional free energy profile. Open and closed populations are 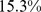, and 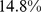, respectively. The remaining conformational space is mainly consisting of three intermediate states whose population is about 70%.

To emphasize the prediction ability of our flexible binding model, we show the free energy profiles as a function of RMSD from the X-ray structure of closed form of diUb (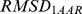) and the distance between the center of mass of Ub units (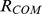) or the distance between the center of mass of I36 hydrophobic patches (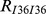) (Fig. 5A and B). The free energy surfaces show a free energy basin around (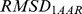,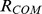) = (0.3,2.6) and (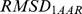,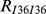) = (0.3,1.7), corresponding to the closed state of K48-diUb (labeled by “C”). The minimal value of 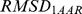 is 0.17 nm (see Table 3 in Text S1). This indicates that the closed state of K48-diUb was validated by our model. In addition to the closed basin of K48-diUb, an open basin is present (labeled by “O”). Remarkably, there are three free energy minima between open and closed basins. These minima represent intermediate states during the conformational change of opening and closing K48-diUb. It is worth noting that these intermediate states are not related to the X-ray structures of compact state of K48-diUb (PDB 1TBE, 3NS8, 3AUL) as indicated by Fig. S10. However, we found that one of these intermediate states (labeled I3) similar to the compact structure (PDB 2PE9) which was determined by NMR spectroscopy [34]. The compact X-ray structures might be resulted from the crystal packing forces, therefore are unstable in solution. Indeed, the theoretical prediction of intermediate states is supported by the NMR data from Fushman laboratory [33]–[35]. They found that K48-diUb in solution rapidly exchanges between at least three major states, including an open state, a closed state and an intermediate state which is hard to be structurally characterized.

Let us go back to the debate on conformational equilibrium of K48-diUb. Considering that there are strong evidences of the presence of intermediate states from simulations and NMR experiments, it is reasonable to construct a simple three-state model in which K48-diUb in solution switches its conformations between open, closed and compact states. The compact state has well-defined interface like closed state. But the difference lies in that in compact state at least one of I44 patches is solvent-exposed and available to be recognized by Ub partner proteins. Subsequently, the three-state model was employed to explain the conformational dynamics of K48-diUb.

To clarify or explain the contradiction between Hirano's and Varadan's measurements, we first have to make clear about the assumptions they adopt in obtaining the conformational population based on experimental data. In fact, Hirano's measurement was performed by comparing chemical shifts of wild-type K48-diUb with that of monomeric Ub as an open state and that of cyclic K48-linked diUb as a closed state. Because the cyclic K48-diUb has two iso-peptide bonds between Ub units which lock the bound conformation fully at closed form, using its chemical shifts as a reference will unavoidably overestimate the actual population of open state in solution. To check this speculation, we measured the population of all three states as shown in one-dimensional free energy profile in Fig. 5C. It shows that the populations of closed and open states are similar to each other at around 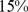. The remaining states occupy 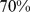 of the whole conformational space. This part of conformational space mostly consists of the compact conformations and contains multiple intermediate states. Considering that both Hirano's and Varadan's measurements were based on a two-state assumption, then, our simulation data can give an excellent explanation on the discrepancy between them. That is, the former was the result obtained from the closed population versus non-closed population, yielding especially high value for open population. Whereas the latter was obtained by monitoring the non-open population versus open population, resulting in a very high value for closed population. Although Hirano et al. argued that using K48C mutant of diUb in Varadan's measurement possibly affected the conformational equilibrium, Varadan et al. tested the K48R mutant and showed no change in the chemical shifts [26]. In other words, from our theoretical prediction, we believe that the differences between experimental observations are more likely caused by the inherent complex multi-state functional landscape rather than artificial errors.

Above all, we predicted a multi-state functional landscape for K48-diUb and found that the multi-state model can be used to well reconcile the seemingly contradictory experimental measurements about the conformational equilibrium.

### Comparison of the functional landscapes of M1-, K48- and K63-diUbs

K63-diUb has been considered to be a typical Ub chain like the classical K48-linked polyUb [4]. In contrast to the essential role of protein degradation of K48-linked polyUb, the function of K63-linked polyUb was found to be linked to numerous nondegradative signaling processes [21]. Several groups have attempted to illuminate the function discrepancy between K48 and K63 linkage from the structural view by using NMR, X-ray crystallography, and SAXS [7], (see also Table 1 in Text S1).

The conformational dynamics of K63-diUb and M1-diUb was explored by the CGK63 model (two Ub monomers linked by a K63-G76 isopeptide bond, see Table 2 in Text S1) and the CGM1 model (two Ub monomers linked by a M1-G76 peptide bond, also denoted as linear model), respectively. Fig. S11 shows the free energy profiles of K48-diUb, K63-diUb and linear diUbs as a function of 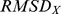 (where X represents the X-ray structures of PDB 3H7P, 3DVG, 3AXC, 2W9N) and 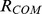. It indicates that the free energy landscapes of K63-diUb and that of M1-diUb are almost identical with each other, however significantly distinct from that of K48-diUb. This implies that K63- and linear diUb share a highly similar functional landscape which is significantly different from K48-diUb. In fact, the spatial position of K63 and M1 is adjacent in the structure of Ub unit (the distance between their separate 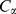 atoms is 0.54 nm), making their constraining effects on conformational space of diUb similar. Beyond the topology similarity, the difference between the two linkage types might lie in the chemical properties of the linkages because M1-linked polyUb is linked by peptide bond rather than isopeptide bond. This is supported by the fact that both K63 and M1 linkage types can recognize the same receptor proteins having ubiquitin-binding domain, but only M1-linked Ub chains rather than K63-linked chains can be specifically cleaved by some deubiquitinase enzymes [7]. The results also support the notion that similar protein topology inclines to generate similar energy landscape [40].

Moreover, the conformational distribution summarized in Table 1, shows that the populations of open conformation for K48-, K63- and linear diUb are about 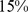, 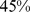 and 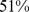, respectively. It also supports the finding that the conformational space of K63- and M1-diUbs is more extended than that of K48-diUb. This is consistent with previous NMR and SAXS observations [7], [38] as well as the recent single molecule fluorescence resonance energy transfer (smFRET) data [41].

Given the excellent qualitative consistence between previous experiments and our simulations, we have to note that the populations of conformational states from our simulations have differences with that from recent smFRET measurement [41]. The smFRET analysis suggested that the compact state of K63-diUb and M1-diUbs is 70% and 75%, respectively. It is worth pointing out that, quantitative analysis in the experiment was based on the dye distance between the N-terminal of distal Ub and the C-terminal of proximal Ub. In order to draw direct comparison from the smFRET data, we employed the 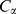 distance between M1 of distal Ub and G76 of proximal Ub (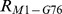) as the order parameter to obtain the conformational distribution (Fig. S12). The results from the simulations suggested that conformational distribution from this order parameter is not sufficient to reflect the complicated energy landscape of polyUb chains. Especially for K48-diUb, the predicted intermediate states are completely hidden on the one-dimensional free energy profile as a function of 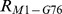 (Fig. S13). In fact, our recent work suggested that the multi-state functional landscape of adenylation kinase also cannot be well characterized by a single order parameter [28]. On the basis of this, we strongly recommend to use multiple order parameters rather than one to monitor the functional dynamics of a protein, especially for one with a possible complex landscape. Experimentally, simultaneous measurement of multiple pairs of fluorescent dyes or multi-color FRET is feasible [42].

Nevertheless, the finding that M1-diUb has slightly more open conformations than K63-diUb from simulations is consistent with that from smFRET experiments. Remarkably, the difference between the population of M1-diUb and that of K63-diUb (5%) measured by smFRET is in reasonable agreement with the population difference measured by simulations (6%). This indicates our simulations not only exactly captured the small population difference but also their relative tendency to form compact conformations, that is, first K48-, then K63- and lastly M1-diUb.

### K11-diUb vs. K48-diUb

While polyUb chains with K48 and K63 linkages as canonical Ubs are the best studied, the K11 linkage is the most prominent among the left six atypical or non-canonical types and its relative abundance in yeast was found to be up to 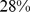 which is comparable to the level of K48 linkage [43]. Note that the high abundance is not validated in higher eukaryotes [13]. Several reports have identified its role in cell cycle regulation as an efficient proteasomal degradation signals like K48-linked chains [11], [44], [45]. This poses a question: How the two proteasomal degradation signals are distinguished by proteasome?

**Figure 6:**
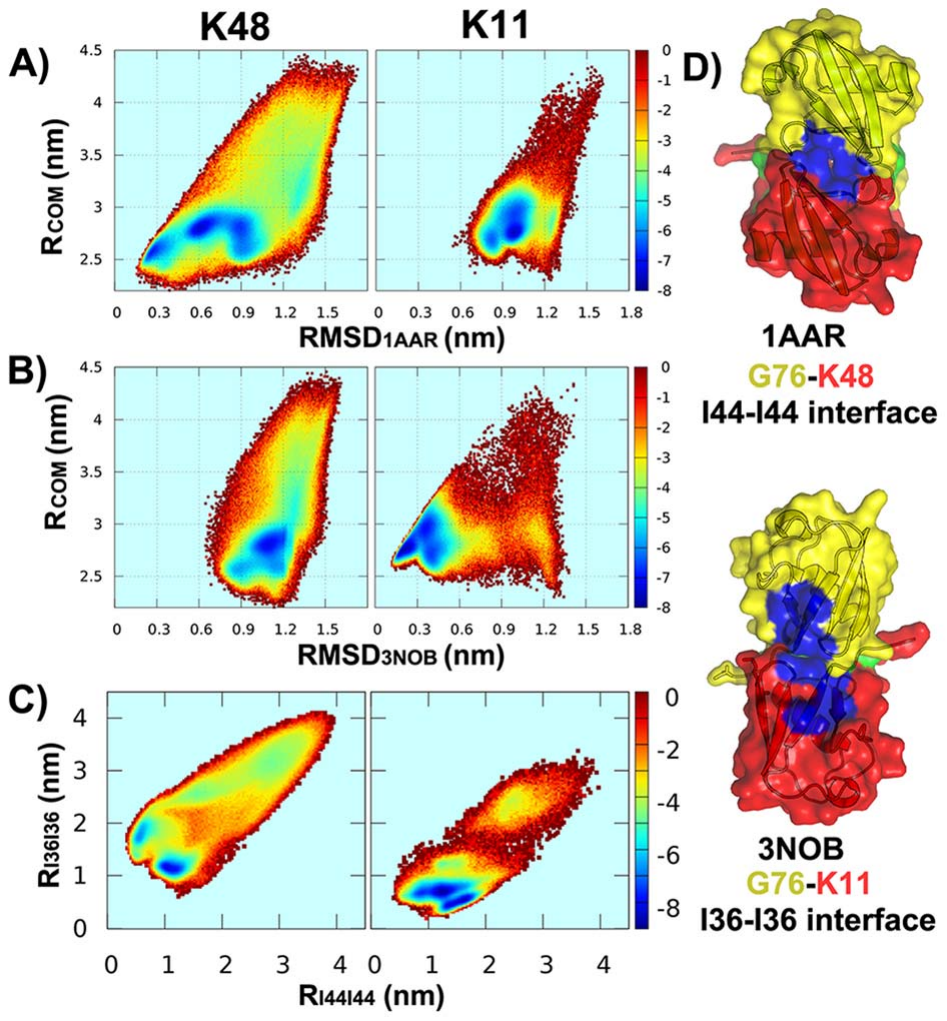
A significant distinct free energy landscape of K11-diUb from that of K48-diUb. (A) Free energy profiles as a function of 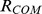 and RMSD from the X-ray structure of the closed form of K48-diUb (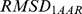). (B) Free energy profiles as a function of 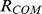 and RMSD from the X-ray structure of the compact form of K11-diUb (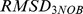). (C) Free energy profiles as a function of the distance between I36 hydrophobic patches (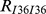) and the distance between I44 hydrophobic patches (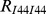) (D) Distribution of average interfacial contacts along the residue index of proximal Ub (black) and distal Ub (grey). Note that the results of K48-diUb model and K11-diUb model correspond to left and right subfigures, respectively.

A partial answer to this question may be derived from the view of energy landscape. Fig. 6A and B show that the free energy surfaces as a function of 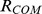 and RMSD from the X-ray structure of the closed form of K48-diUb (PDB 1AAR) and the compact form of K11-diUb (PDB 3NOB), respectively. Note that, the conformational dynamics of K11-diUb was investigated by the CGK11 model (two Ub monomers linked by a K11-G76 isopeptide bond, see Table 2 in Text S1). We can see that there is a free energy basin formed around the conformational region with RMSD less than 0.3 nm on the free energy surface F(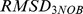,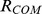). It indicates that the experimental compact structure of K11-diUb is perfectly validated by our model. By comparison with the free energy surface of K48-diUb, it further suggests that the functional landscapes of K48-diUb and K11-diUb have strikingly difference with each other. This is also supported by the free energy surface (Fig. 6C) projected on two order parameters independent of native structures. They are the distance between I36 hydrophobic patches (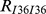) and the distance between I44 hydrophobic patches (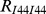). Fig. 6C shows that, K11-diUb mostly populates at the conformational region with 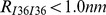 while K48-diUb at the region with 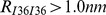.

Moreover, the distribution of interfacial interactions between distal unit and proximal unit in K11-diUb is also distinctly different from that in K48-diUb, as shown in Fig. 3H. In contrast to K11-diUb whose interfacial interaction distribution seems to be relatively symmetrical as the distribution of free Ub monomers, the distribution of K48-diUb is obviously asymmetrical. It is interesting and worthnoting that K11 is the only one whose interface symmetry is not significantly broken by the formation of linkage between Ub units (see Fig. 2). For K48-diUb, the “hot spot” residues (corresponding to the residues contributing more than one average interfacial contact, see Fig. 3) in distal Ub comprises T7-G10, Q40, R42, I44, G47, H68 and V70-G76, while in proximal Ub contains K6-G10, R42, I44, A46-Q49, H68, V70-L73, G75 and G76. By comparison, the hot spot residues at the interface between distal of K11 and proximal Ubs are comprised of T7-K11, E34-P37, Q40 and V70-G76.

The distributions of hot spot residues enable us to shed light on the binding patterns between Ub units. Our results suggest that the I36 patches (consisting of L8, I36, L71 and L73) on both distal and proximal Ubs play a significant role on the formation of the interface of K11-diUb but not of K48-diUb, while the I44 patches (consisting of L8, I44 and V70) play a more important role on the interface of K48-diUb. In other words, we found that unlike K48-diUb in which the I44 patches can be totally buried in its closed form, for K11-diUb its I44 patches are scarcely shielded so as to be accessible to protein partners containing ubiqtitin-binding domains. Taken together, our results demonstrate that the functional landscapes and binding patterns of K11-diUb and K48-diUb have significant discrepancy. This may explain why the two share-function polyUb chains can be distinguished by proteosome, and why some proteins specifically interact with K11-linked Ubs but not with other polyUb chains [13], [45].

### Other atypical linkages: K6, K27, K29 and K33

Less is known about the remaining four atypical linkages involving K6, K27, K29 and K33 which are more rare in cells [7]. Especially for the three linkages K27, K29 and K33, their ubiquitin lysine residues are very close with each other not only in sequence space but also in configuration space (the distances between 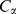 atoms are in range of 0.5–0.8 nm), making them difficult to be measured by experimental tools, such as mass spectroscopy [46]. Currently their functional roles are not well-established, but there are evidences suggesting K29 and K33 as well as K6 linkages might play many non-proteolytic roles and K27 is important to mitochondrial biology [4].

**Figure 7:**
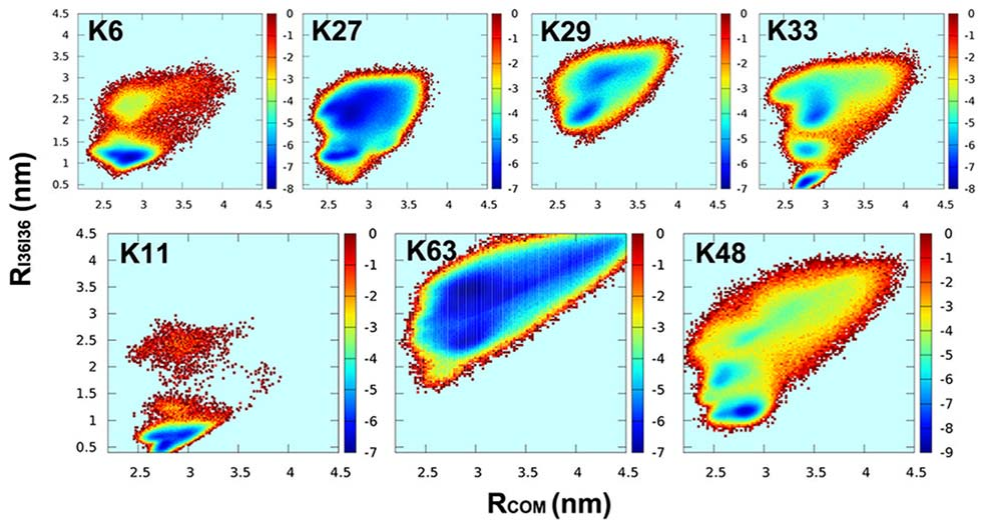
Functional landscapes of K6-, K27-, K29- and K33-diUbs. Upper subfigures are the free energy profiles of K6, K27, K29 and K33 linked diUbs plotted as a function of 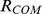 and 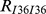. Lower subfigures are the free energy profiles of K11, K63 and K48 linkages, shown as references. They represent compact, open and the multi-state functional landscapes, respectively.

Fig. 7 shows the free energy surfaces of K6, K27, K29 and K33 linkages, respectively. In order to predict the conformational space, we used two order parameters 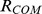 and 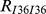 because they are not dependent on specific structures like RMSD. The free energy surfaces of K11, K63 and K48 are also shown as references of compact, open and multi-state landscape, respectively. By further analysis of their conformational spaces, we found that although K6-diUb and K11-diUb have highly compact conformational spaces, their compact states are different. The conformational space of K6-diUb is mainly contributed by compact structures with an I36-I44 interface, while that of K11-diUb by compact structures with an I36-I36 interface. We also found that, for both K27-diUb and K29-diUb, their conformations are more compact than that of K63-diUb and more open than that of K11-diUb, but are distinct from each other. These conclusions are also consistent with the aforementioned analysis of the conformational distributions (Table 1 and Fig. 6). Remarkably, there are multiple free energy basins on the functional landscape of K33-diUb (see also Fig. S14), corresponding to multiple functional states. By further inspecting the structures of these functional states, we identified two of them are similar to the compact structures of K6-diUb (PDB 2XK5) and K11-diUb (PDB 3NOB). This multi-state feature of K33-diUb is highly similar to that of K48-diUb. To our best of knowledge, our work provides the first theoretical evidence of the presence of multiple functional states on the functional landscape of K33-diUb.

Interestingly, despite the close sequence and spacial positions between the three linkage residues K27, K29 and K33, the resulting functional landscapes have dramatic differences between each other. Our theoretical prediction can be validated by future experiments. This prediction further makes us to argue that, Ub as a small globule protein (with 76 residues) has been evolutionarily selected by nature, endowing it with the stable foldability and subtle binding specificity. So even a small shift of topological constraint can result in a large change of interfacial interactions. The topological sensitive of resultant functional landscape can be well explained by the highly designed local environment on the surface of Ub. From microcanonical perspectives, the topological constraint of covalent linkages is purely entropic, and dramatically reduces the conformational search space. However, the result could be the changing of the gap and roughness of the intrinsic landscape [47]. This is purely due to the limitation resulting the selection of local interactions (local in sequence and space) from the topological constraint. Thus, the topological constraint of all eight linkages provides a way for breaking the binding symmetry and reaching the functional specificity.

## Discussion

The post-translational modification of proteins in the cell is an important theme in molecular and cell biology. Different from other post-translational modifications, such as glycosylation, methylation and phosphorylation, ubiquitination provides a more versatile cellular signaling mechanism [7]. This is mostly contributed by the possibility of Ub units to form different polyUb chains through eight different linkages (M1 and all seven lysines). Linking multiple Ub units in one chain not only strengthens the Ub signal, but also provides further differential new signals through the formation of numerous conformations [32]. The canonical ubiquitin chains, such as K48- and K63-linked chains, have been well characterized, both structurally and functionally. Despite of this, other linkages are less understood and all linkage types have to be studied in order to obtain a full picture of ubiquitin code [48].

**Figure 8:**
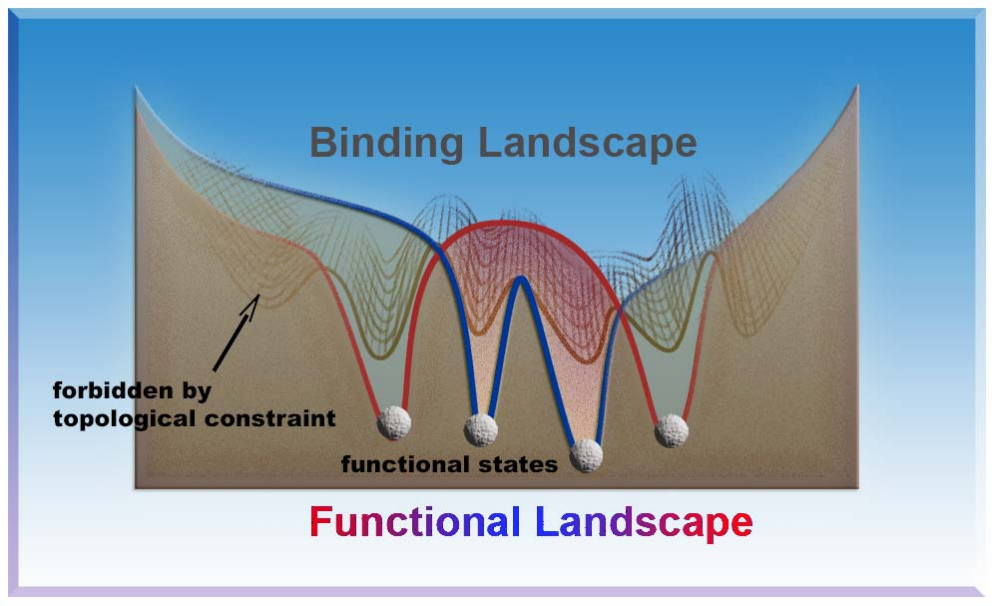
Schematic picture that the functional landscapes of different diUb chains are selected from the same binding landscape of two Ub monomers by corresponding topological constraint. The binding landscape (grey) is a description of binding between two free Ub monomers, while the functional landscapes are a description of binding between two Ub units with an additional topological constraint, e.g. a peptide bond formed between proximal Ub and distal Ub. Two typical different topological constraints are represented by red and blue, respectively. Our simulation results reveal that the binding landscape contains most of functional states of diUbs with different linkage types. And part of these functional states are further stabilized by the corresponding linkages. Such as, the functional landscape of K48-diUb has a deeper basin of the I44-I44 conformational state which is also present on the binding landscape. But other functional states, such as compact conformation of K11-diUb and K63-diUb, cannot be sampled on the functional landscape of K48-diUb. In other words, these conformational space is forbidden as a consequence of specific topological constraints (grey and dark regions). The topological constraint limits the conformational space and selects the local interactions for realizing the corresponding biological function. Therefore, the topological constraint provides a way for breaking the binding symmetry and reaching the functional specificity.

In the present work, we explored the binding landscape of two free Ub monomers as well as the global functional landscapes of all eight linkage types by theoretical modeling. The results lead to a number of significant conclusions. In particular, the simulations from the free Ub model give a remarkable finding that most of the compact structures of covalently bonded diUbs resolved by X-ray or NMR pre-exist on the binding landscape of free Ub monomers. Additionally, all these experimental structures were validated by the corresponding linkage models. It is worth noting that our flexible binding model does not contain any prior knowledge of native protein-protein interactions in these structures. Therefore the pre-existence of native states of polyUb chains on the binding energy landscape of free Ubs is rather surprising. It also suggests the prediction ability of our flexible binding model despite at coarse grained level. Moreover, this finding leads us to speculate that the well-folded architecture of Ub monomer has embodied the information of forming polymerized functional structures on its binding energy landscape. The corresponding functional landscape of diUbs with a specific linkage type is further generated on the basis of the binding landscape on which functional states are selected by topological constraints as a form of covalent linkages between Ub monomers, as shown in Fig. 8. It is important to note that only part of functional states on the binding landscape can be sampled on the corresponding functional landscape of a specific linkage type. Other part of functional states can only be sampled by the corresponding linkage types. In other words, most of conformational space of the binding landscape is forbidden on the functional landscape as a consequence of specific topological constraints. The conformational restriction arising from the topological constraint may account for the symmetry breaking of the interfacial interactions. In addition, it also can reduce the entropy of the functional landscape so as to facilitate the fast searching of functional states. The specific topological constraint also selects the local interactions for realizing the corresponding biological function. Therefore, the topological constraint provides a way for breaking the symmetry and reaching the biological specificity. Moreover, it is likely that forming a covalent linkage between two Ub units does not induce the appearance of a new functional state but instead just shifts the population of pre-existing states on the binding landscape. This notion is similar to the “conformational selection” scheme [49], a mechanism which is extensively used in the description of the conformational change of a protein induced by ligand binding [28], [50].

In summary, there are four points revealed by theoretical data consistent well with experimental data: (1) Intermediate states exist on the functional landscape of K48-diUb, besides the known open and closed states. (2) K63- and linear diUb share a highly similar functional landscape which is significantly different from K48-diUb. (3) M1-diUb has slightly more open conformations (about 6%) than K63-diUb. (4) The functional landscapes and binding patterns of K11-diUb are significantly different from that of K48-diUb.

Furthermore, our simulations predict several points that may be validated by future experiments: (1) Hydrophobic interactions play a more dominant role in K6-, K11-, K48- and K33-linked diUbs. By contrast, electrostatic interactions are more important to the association of Ub units in K27, K29 and K63 as well linear linkages. (2) The conformational distribution of K6- and K11-diUbs is highly dependent on pH like K48-diUb. Decreasing pH will increase their open conformations. (3) K33-diUb has a multi-state landscape where two functional states are similar to the compact structures of K6-diUb as well as K11-diUb. (4) The symmetry of interfacial interactions is not significantly broken in K11-diUb. (5) The functional landscapes among K27-, K29- and K33-diUbs have dramatic differences between each other despite the close sequence and spacial positions between the three linkage residues.

While there are relatively abundant structures of K48-diUb, as a number of snapshots of its highly dynamic conformations, have been captured, people speculated that other linkage types also have other conformations which could be adopted besides the limited available structures [11]. This speculation was validated by our present simulation work. Not only three intermediate states are predicted to be present on the functional landscape of K48-diUb, other diUb chains, such as K33-diUb, are suggested to have several unprecedented functional states. Inspired by the multi-state landscape, we employed a simple three-state model to well reconcile the seemingly contradictory experimental data on the conformational equilibrium of K48-diUb. The multi-state conformational equilibrium is believed to play an important role in the recognition of numerous proteins containing ubiqtitin-binding domains.

Despite of ever increasing amount of three dimensional structures of protein-protein complexes and multi-domain proteins resolved by biophysical tools, especially X-ray crystallography and NMR spectroscopy, there are still big gaps between the number of experimental structures deposited in the Protein Data Bank and the number of predicted binary interactions between human proteins [51]. From this point, our coarse-grained protein-protein interaction model provides the computational community with a flexible binding tool necessary to predict the complexed structures of proteins or protein domains. More importantly, our method has the ability to give global information (including number of functional states, their populations and free energy barriers between each other) on the protein system. Given the exciting success on the implication of this method to polyUb chains, we will extend our flexible binding model to more protein systems and expect to exhibit the prediction ability in the future.

## Methods

Traditional structure-based model (SBM) cannot be applied directly in the present work to predict the conformational dynamics of polyUb chains because several linkage types (including K27, K29 and K33) still have not been structurally determined at present. Here, we developed a new model by extending the SBM to address the specific problem of polyUb chains. In our model, each Ub unit is built based on coarse-grained SBM in which each amino acid residue is represented by two beads (one for backbone, the other for sidechain) and the interactions within Ub unit are structure-based. To further describe the association between Ub units, electrostatic forces and hydrophobic effects are introduced to represent the non-native interactions. In other words, the interactions within Ub units are structure-based as traditional SBM which requires the structural information of Ub monomer, but the interactions between Ub structural units are physics-based without the need of any information of polyUb chains. Therefore, this model not only can keep the computational speed advantage inherited from coarse-grained SBM but also has the potential to predict the binding mechanism of Ub units in the context of polyUb chains. The hamiltonian energy function of our model can be given by the expression:
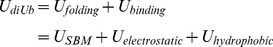
Where folding potential 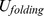 is equal to 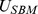, which is the force field of SBM to describe the conformational fluctuation of Ub units around folded basin. While the binding potential 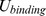 can be decomposed into 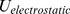 and 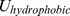 terms, which are the potential of electrostatic interactions and hydrophobic forces, respectively. The SBM term 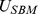 is divided into backbone and non-bonded terms. The latter term can be further partitioned into two components consisting of an attraction term and a repulsive term. Note that contact and dihedral potentials were rescaled as previously described [28]. The detailed description of 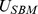 can be found elsewhere (see ref. [24], [52]).

Simulations were performed with Gromacs 4.0.5 [53] at temperature T = 50 (close to but lower than folding temperature). This temperature is used throughout the work, except where specified. It is worth pointing out that the temperature in the simulations is difficult to exactly correlate with actual temperature in Kelvins due to the coarse-grained representation and the use of reduced units. A time step of 0.0005 time units was used and the simulation was coupled to a temperature bath via Langevin dynamics with a coupling time of 1.0. To guarantee the convergence of the sampling as well as the robustness of the results, we performed eight independent long simulations with the same parameter sets for each model. Each simulation has 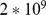 time steps. Subsequently, these independent simulations were collected together to calculate the free energy surfaces and population distributions of conformational states. In fact, the single independent simulation was already well converged as shown in Fig. S15.

### Structure-based model for Ub unit: Coarse graining and reference structure

Each amino acid in polyUb chain is represented by one bead or two beads with or without charge which is dependent on its property. One bead named 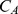 in our model is located at the position of the 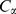 atom. The other bead named 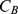 is located on the center of mass of the sidechain atoms (with exception of Gly). The 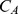 bead and 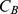 bead are used to coarse grain the atoms of backbone and that of sidechain, respectively.

We used the structure of Ub monomer (PDB: 1UBQ) resolved by X-ray crystallography [54] as the reference conformation for structure-based model (SBM). It is important to point out that this structure of Ub monomer is the only structural information as an input of the present model. In addition, we found that the C-terminus of Ub monomer is quite flexible, as shown in Fig. S16. Such flexibility reflected in the free model is naturally implemented into all of the linkage models, because all models share the same structure-based potential for Ub units.

### Beyond SBM: Interfacial electrostatic and hydrophobic interactions

The electrostatic potential was represented by the Debye-Huckel (DH) model in which the details can be found in our previous works and elsewhere [24], [28], [55]. In this study, we set the dielectric constant 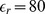 and focus on physiological salt concentrations of 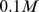 (lead to the Debye screening length 10 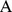) which are consistent with the experimental condition [22]. All charged residues were assigned charges according to their electrostatic properties at neutral pH 7. Then, 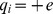 for Lys and Arg, 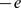 for Asp and Glu, and 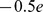 for His, where e is the elementary charge.

To account for the hydrophobic interface formation, we introduce nonnative hydrophobic interactions by the expression which is borrowed from Chan and coworkers' model [56]:
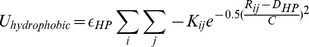
Where 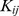 is the hydrophobicity strength between beads i and j, 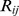 is the distance between bead i and j and 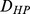 the optimal distance to form interfacial hydrophobic interactions between Ub units. After testing with an extensive set of 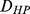
s ranging from 5.0 to 10.0 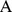, we finally set 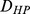 to be 8.0 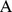 instead of 5.0 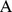 which was used in protein folding study in ref. [56]. Constant C can be used to adjust the basin width of hydrophobic potential but is set to be 1.0 for simplification. 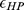 is introduced to modulate the balance between the hydrophobic force and electrostatic force. After the calibration of hydrophobic potential by setting 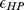 to be 0.92, the binding affinity was estimated to be in an order of magnitude of 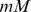 (at 0.1 M salt concentration and 5 mM protein concentration, see the calculation in SI Appendix), in good agreement with experimental measurement [22].

The parameter set of 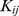 is obtained from our recent works [57], [58] but with a bit modifications by using the expression 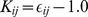 as 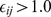. By doing so, we can shift the parameter set of hydrophobic interactions into the region between 0 and 1. Note that we set the hydrophobic strength to be zero as 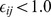 so as to avoid overestimating the electrostatic interactions which have been separately modelled by DH potential. In the original work [59], 
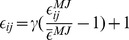
. 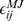 is the original Miyazawa-Jernigan (MJ) potential, 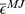 is the mean value of the entire set of MJ weights in this protein system, 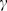 is a variable to modulate the strength of energetic heterogeneity which reflects the sequence discrepancy, it is set to be 1.0 corresponding to the sequence-flavored model [59]. In other words, 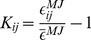 in the present work. 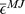 was found to be −3.4 and −3.7 in a coupled folding and binding system (histone chaperone and histone H2A.Z-H2B) [58] a multi-domain protein (Y-Family DNA Polymerase) [57], respectively. Here, we used the latter value because it can normalize 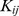 (the lowest MJ weight −7.37 is about twice of −3.7). The final parameter matrix of hydrophobic interactions is shown in Fig. S17.

### Modelling of different linkage types

To model the diUb chains with different linkage types, we introduced the bond-stretching potential in the form of a harmonic potential,
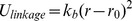
where k_b_ is the spring constant, r is the bond length and 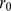 is the reference bond length. Here we set 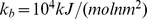, 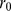 is set to be 0.4nm for an isopeptide bond (for all seven lysine linkage types) and 0.38 nm for a peptide bond (for M1 linear linkage type).

## Supporting Information

Figure S1
**Compact structures of diUb chains with different linkages can be sampled by the free Ub model in which the two Ub monomers are not connected by a covalent bond.** The free energy surfaces were plotted as a function of the centroid distance between Ub units (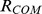) and the RMSDs from available structures resolved by X-ray crystallography and NMR (listed in Fig. 1 in main text). The x axis corresponds to 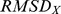 (in unit of nm, X represents the PDB code). The y axis corresponds to 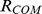 (in unit of nm). The grey regions highlight the conformational space near the experimental structures. It indicates that Ub monomers in the free form can sample the conformational regions involving the experimental structures of diUbs with different linkages. The minimal RMSDs to experimental structures are summarized in Table 2 in Text S1. Note that these free energy profiles were derived from a single free Ub simulation.(PDF)

Figure S2
**A control result to show that free Ub monomers never sample all possible conformations.** The MD trajectories of the free Ub model were projected to the two-dimensional free energy surface as a function of 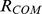 and RMSD to a dimeric conformation extracted from chain K and chain L of the crystal structure with PDB code 2XEW (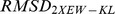). Its structure is a result of crystal packing forces, thus represents a “wrong” structure. The free energy surface clearly indicates that the free Ub model doesn't sample such conformation. In contrast, the free Ub model can sample most of the “right” compact conformations. This indicates that the assembly of free Ub monomers into similar functional states of diUbs is far beyond random events.(PDF)

Figure S3 **Interfacial contact maps of the free Ub model and the eight linkage models.** (A) Free Ub monomers. (B) K11-diUb. (C) K27-diUb. (D) K29-diUb. (E) K33-diUb. (F) K48-diUb. (G) K63-diUb. (H) Linear diUb.
(PDF)

Figure S4
**Estimation of entropic and enthalpic contributions of a bonded constraint by the formula** 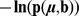 **.** 
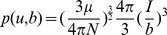
 is the average probability to form a bond/contact constraint with 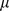 contacts/bonds already present, where N is the residue number, b is the persistent length of a peptide chain, and I is the length of the bonded constraint. For our diUb models, N = 152 and I = 4.0 Å.(PDF)

Figure S5
**Entropy-enthalpy compensation analysis by decomposing free energy F(x) into** 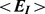 **(x) and TS(x).** Here x is 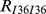.(PDF)

Figure S6
**Entropy-enthalpy compensation analysis by decomposing free energy F(x) into** 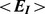 **(x) and TS(x).** Here x is 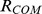.(PDF)

Figure S7
**Entropy-enthalpy compensation analysis by decomposing free energy F(x) into** 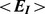 **(x) and TS(x).** Here x is 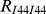.(PDF)

Figure S8
**Hydrophobic interactions** 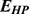 **and electrostatic interactions** 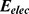 **at the interface between Ub units.** The results are from the simulations by the free model. (A) Free energy profile as a function of 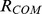 and interfacial 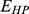. (B) Free energy profile as a function of 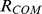 and interfacial 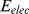. (C) Free energy profile as a function of interfacial 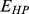 and interfacial 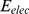. 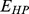 is negatively related to 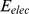. (D) Distribution of 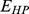 and 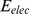 as a function of the distance between I44 hydrophobic patches of two Ub monomers (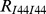). Green color represents the electrostatic interaction distribution and red color represents the hydrophobic interaction distribution.(PDF)

Figure S9
**Criteria for determining the open, closed and compact states are defined according to the corresponding free energy profiles.** It shows the free energy profiles as a function of 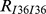, 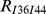 and 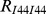, as an example. The peak value of the transition state region was used as the cutoff distance to define these states. Thus, 0.96 nm, 0.93 ns and 0.86 nm are used to define the I36I36, I36I44 and I44I44 substates, respectively.(PDF)

Figure S10 **Three intermediate states revealed on the free energy surface of K48-diUb.**
(PDF)

Figure S11
**The free energy surfaces between K63-diUb and linear diUb are highly similar to each other but significantly different from K48-diUb.** The free energy surfaces 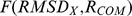 were plotted as a function of 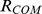 and 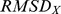. X represents the X-ray structures of 3H7P, 3DVG, 3AXC and 2W9N which are shown above as the open and compact conformations of M1- and K63-diUbs. The x axis corresponds to 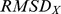 (in unit of nm). The y axis corresponds to 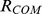 (in unit of nm). The free energy surfaces of K63-diUb and M1-diUb are almostly identical with each other, however significantly distinct from that of K48-diUb. Note that the corresponding linkage models were used to sample the conformational spaces of K48-, K63- and M1-linked diUbs.(PDF)

Figure S12 **Conformational distribution of K63-diUb based on the order parameter similar to smFRET measurement.** It is difficult to monitor the conformational space of K63-diUb via this order parameter. However, the populations of open and compact states are estimated to be 24% and 76%, respectively. This is quantitatively consistent with the smFRET data. Despite of this, it is better to investigate the conformational dynamics of diUb with more than dye pairs because one-dimensional conformational distribution or free energy profile have been suggested to be not sufficient to describe the multi-state landscape of many proteins.
(PDF)

Figure S13 **Evidence that intermediate states are possibly hidden on the one-dimensional conformational distribution or free energy profiles but can be detected by multi-dimensional free energy profiles.** Note that the data is from the simulation of K48-diUb model.
(PDF)

Figure S14 **Functional landscape of K33-diUb.** K33-diUb only can form the similar conformation to the compact state of K6 with I36–I44 interface (PDB 2XK5) but also is able to sample the similar conformation of the compact state of K11 with I36-I36 interface (PDB 3NOB).
(PDF)

Figure S15
**Convergence analysis of single independent simulation.** As a typical simulation, one of the MD trajectories of K48-diUb model was chosen to show the result. The trajectory was projected onto three observables (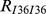, 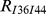 and 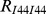), (A) Autocorrelation functions of 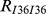, 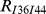 and 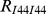. (B) A typical MD trajectory of 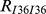. The autocorrelation analysis indicates the correlation time 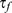 of these observables is about 10 ns. Note that the correlation time measures the length of simulation time required for the trajectory to lose correlation with earlier observables. The ratio of the total simulation time 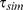 to the correlation time 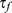 can validate if the simulation has sufficient statistically independent observables, so as to provide an estimation of the sampling quality. Thus, in our simulation, 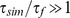 suggests a good sampling. (C–E) Free energy profiles of 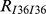, 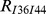 and 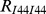 as a function of different simulated lengths ranging from 200 ns to 1000 ns. We can see that the curve of free energy profiles is well conserved after 300 ns. This also indicates convergent results. In addition, the symmetry of interfacial interactions between Ub units also suggests the sufficient sampling of the simulations of the free Ub model.(PDF)

Figure S16 **Comparison between conformational fluctuation of Ub units with experimental temperature factor.**
(PDF)

Figure S17 **The matrix of hydrophobic parameters for different sidechain-sidechain interactions in the protein-protein association model.**
(PDF)

Text S1 **Supporting Methods (Estimation of disassociation constant, Entropy reduction estimation by a polymer theory); Tables 14; Supporting References.**
(PDF)
