# Supplementary figures and images for: PolyUbiquitin Chain Linkage Topology Selects the Functions from the Underlying Binding Landscape
Source: PLoS Comput Biol. 2014 Jul 3;10(7):e1003691. doi: 10.1371/journal.pcbi.1003691 (PMC4081019; doi:10.1371/journal.pcbi.1003691)

R<sub>COM</sub> (nm)

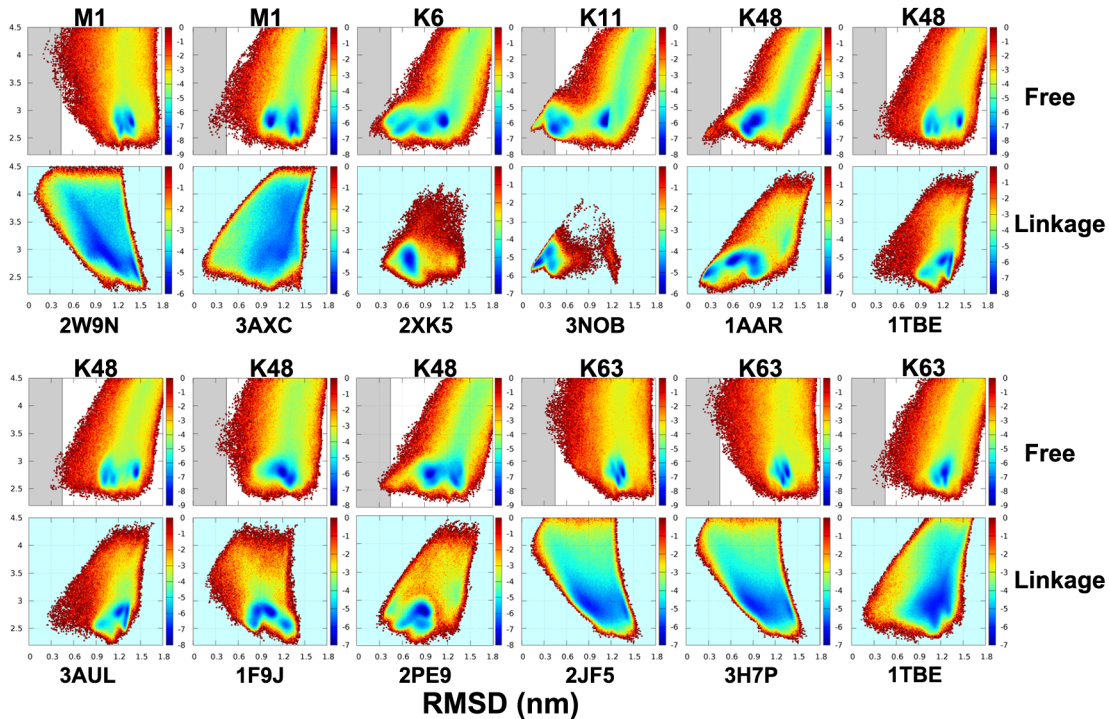

Supplement: Figure S1 — Compact structures of diUb chains with different linkages can be sampled by the free Ub model in which the two Ub monomers are not connected by a covalent bond. The free energy surfaces were plotted as a function of the centroid distance between Ub units () and the RMSDs from available structures resolved by X-ray crystallography and NMR (listed in Fig. 1 in main text). The x axis corresponds to (in unit of nm, X represents the PDB code). The y axis corresponds to (in unit of nm). The grey regions highlight the conformational space near the experimental structures. It indicates that Ub monomers in the free form can sample the conformational regions involving the experimental structures of diUbs with different linkages. The minimal RMSDs to experimental structures are summarized in Table 2 in Text S1. Note that these free energy profiles were derived from a single free Ub simulation. (PDF) [file pcbi.1003691.s001.pdf]

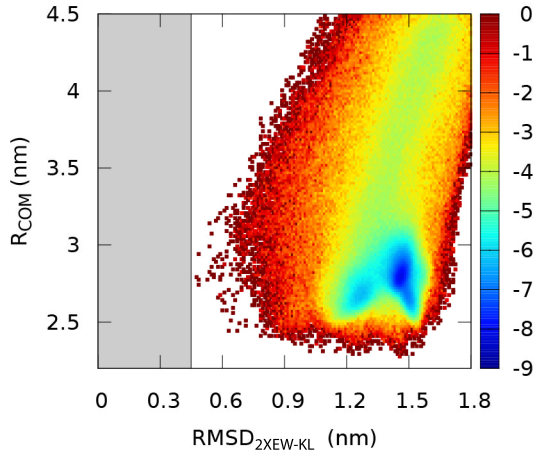

Supplement: Figure S2 — A control result to show that free Ub monomers never sample all possible conformations. The MD trajectories of the free Ub model were projected to the two-dimensional free energy surface as a function of and RMSD to a dimeric conformation extracted from chain K and chain L of the crystal structure with PDB code 2XEW (). Its structure is a result of crystal packing forces, thus represents a “wrong” structure. The free energy surface clearly indicates that the free Ub model doesn't sample such conformation. In contrast, the free Ub model can sample most of the “right” compact conformations. This indicates that the assembly of free Ub monomers into similar functional states of diUbs is far beyond random events. (PDF) [file pcbi.1003691.s002.pdf]

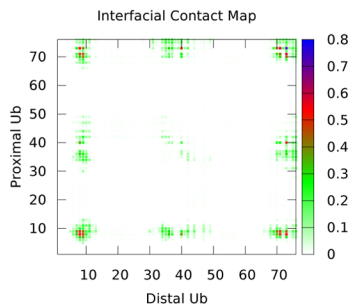

(A) Free

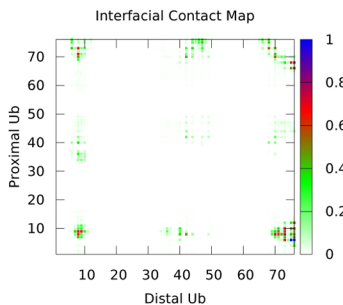

(B) K6

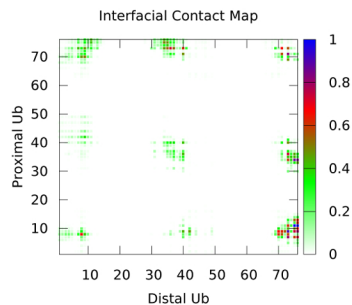

(C) K11

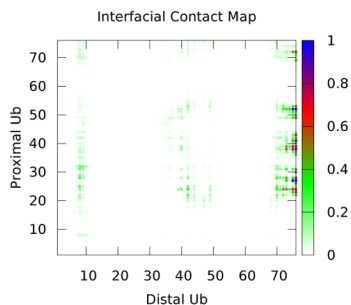

(D) K27

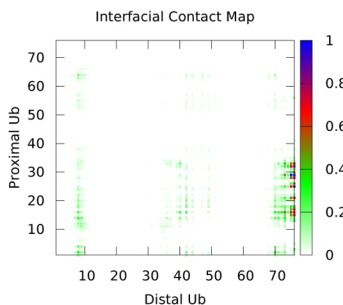

(E) K29

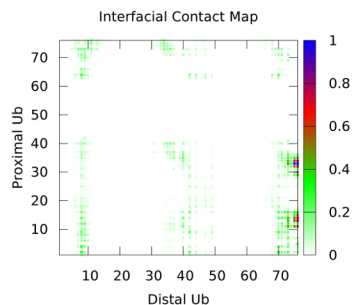

(F) K33

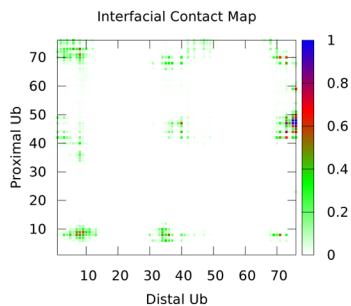

(G) K48

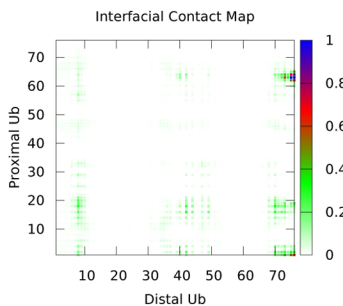

(H) K63

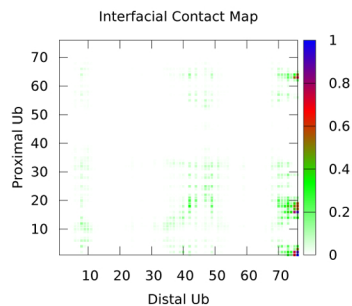

(I) M1

Supplement: Figure S3 — Interfacial contact maps of the free Ub model and the eight linkage models. (A) Free Ub monomers. (B) K11-diUb. (C) K27-diUb. (D) K29-diUb. (E) K33-diUb. (F) K48-diUb. (G) K63-diUb. (H) Linear diUb. (PDF) [file pcbi.1003691.s003.pdf]

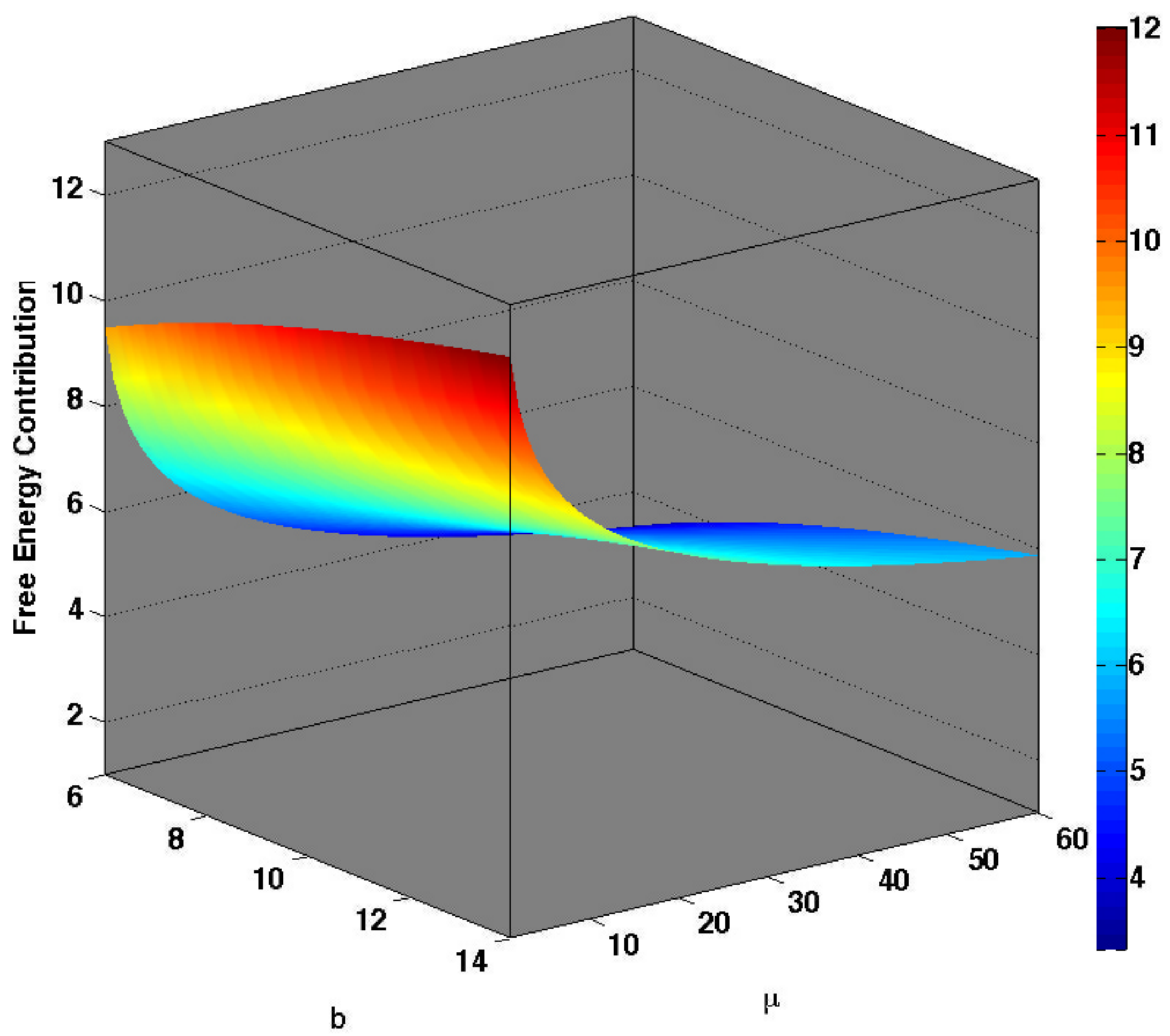

Supplement: Figure S4 — Estimation of entropic and enthalpic contributions of a bonded constraint by the formula . is the average probability to form a bond/contact constraint with contacts/bonds already present, where N is the residue number, b is the persistent length of a peptide chain, and I is the length of the bonded constraint. For our diUb models, N = 152 and I = 4.0 Å. (PDF) [file pcbi.1003691.s004.pdf]

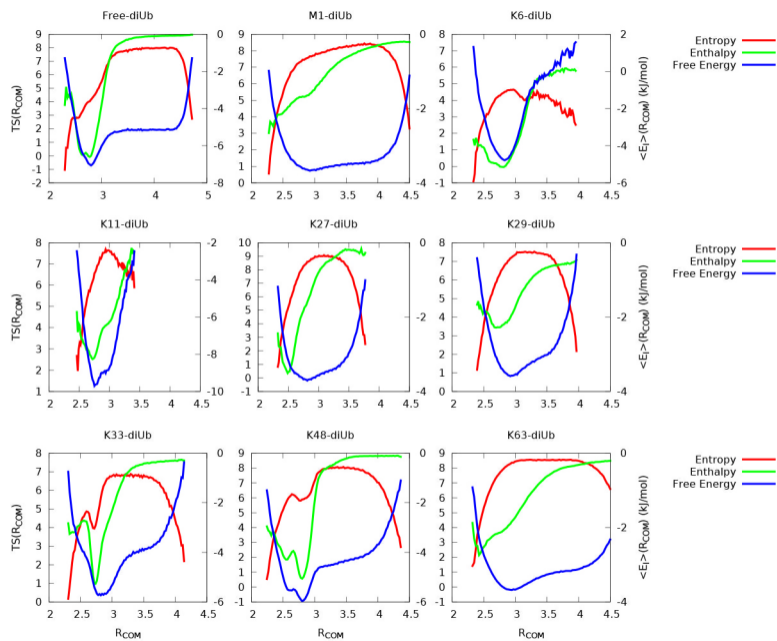

Supplement: Figure S6 — Entropy-enthalpy compensation analysis by decomposing free energy F(x) into (x) and TS(x). Here x is . (PDF) [file pcbi.1003691.s006.pdf]

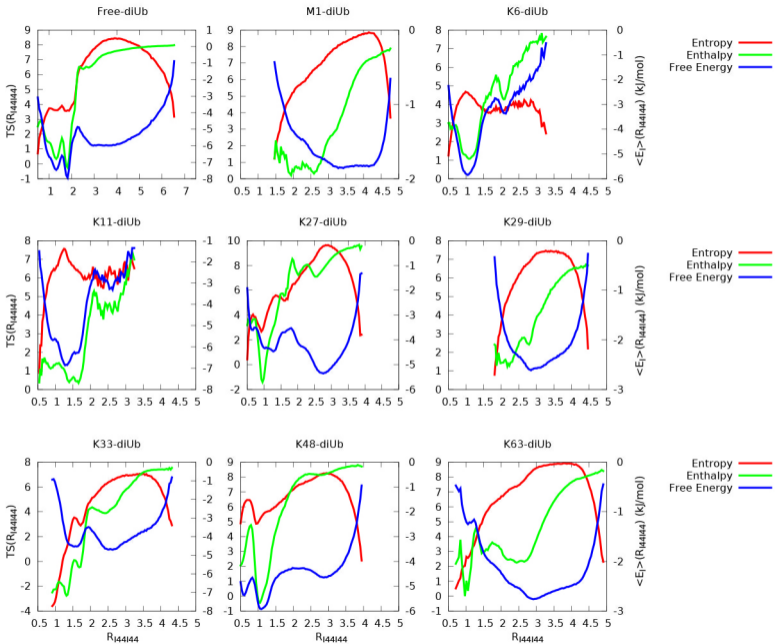

Supplement: Figure S7 — Entropy-enthalpy compensation analysis by decomposing free energy F(x) into (x) and TS(x). Here x is . (PDF) [file pcbi.1003691.s007.pdf]

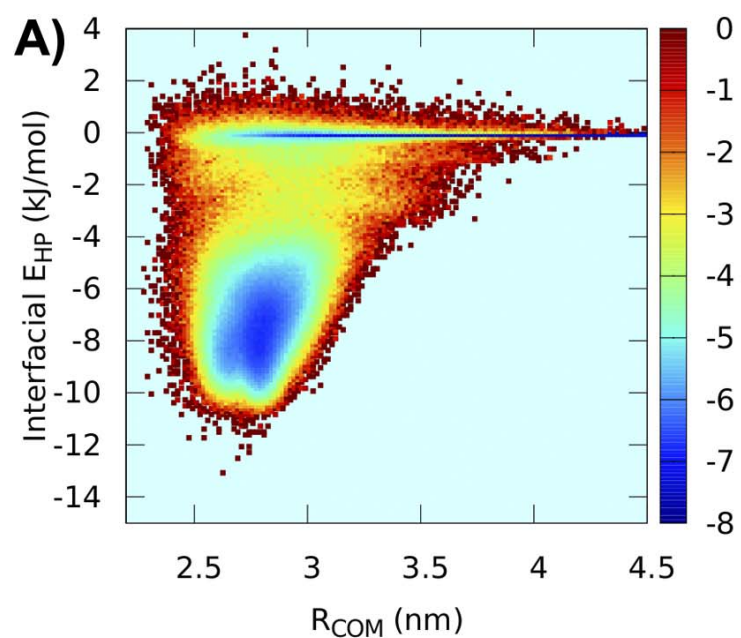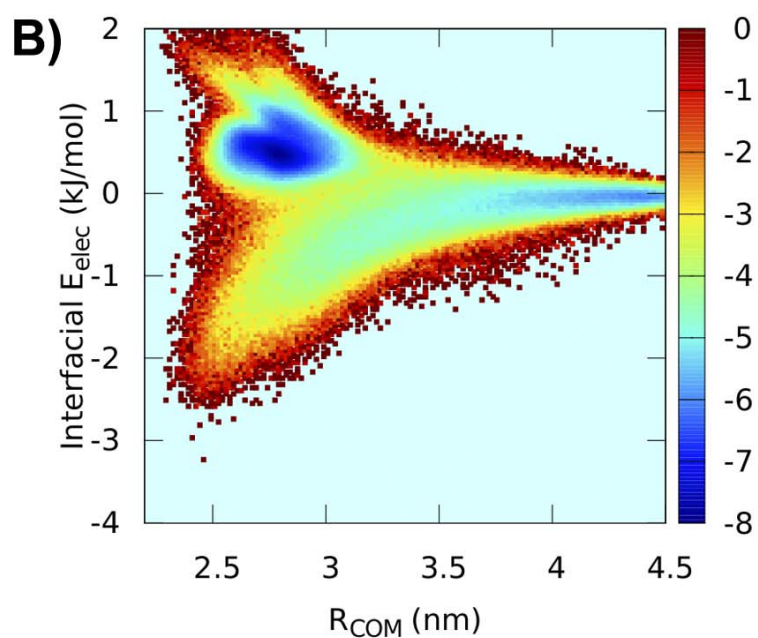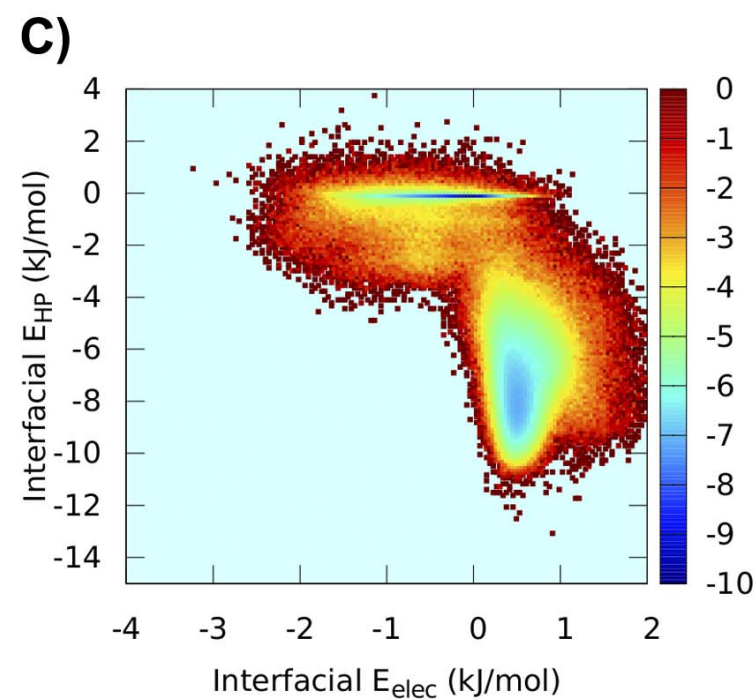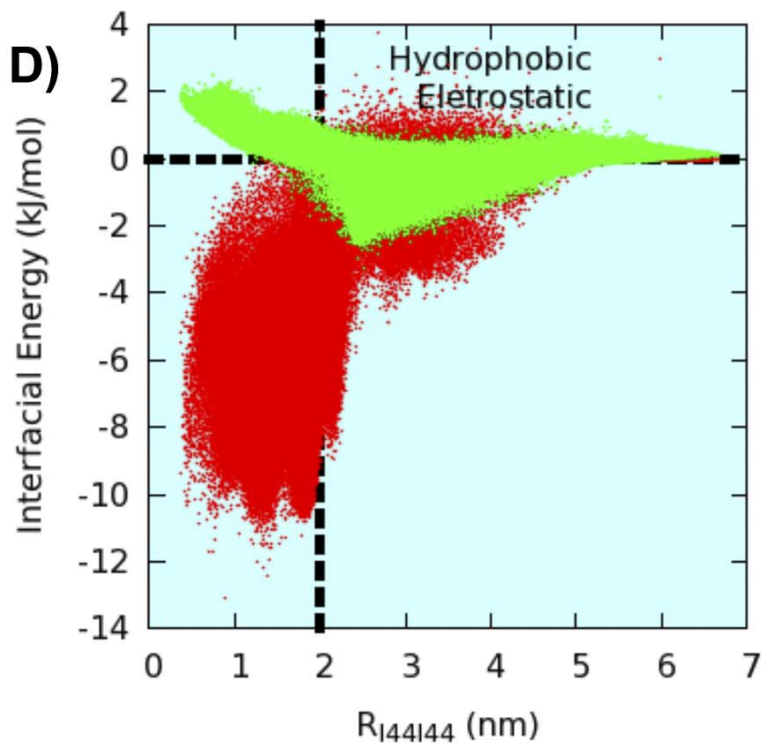

Supplement: Figure S8 — Hydrophobic interactions and electrostatic interactions at the interface between Ub units. The results are from the simulations by the free model. (A) Free energy profile as a function of and interfacial . (B) Free energy profile as a function of and interfacial . (C) Free energy profile as a function of interfacial and interfacial . is negatively related to . (D) Distribution of and as a function of the distance between I44 hydrophobic patches of two Ub monomers (). Green color represents the electrostatic interaction distribution and red color represents the hydrophobic interaction distribution. (PDF) [file pcbi.1003691.s008.pdf]

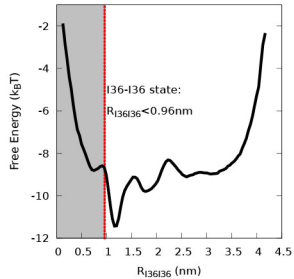

(A)

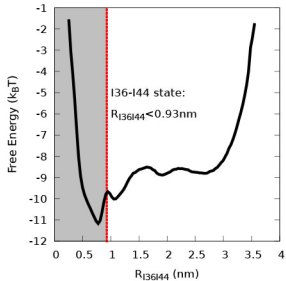

(B)

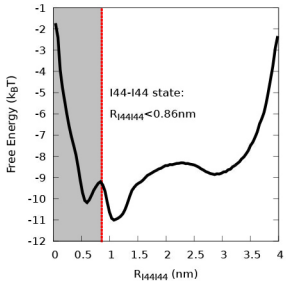

(C)

Supplement: Figure S9 — Criteria for determining the open, closed and compact states are defined according to the corresponding free energy profiles. It shows the free energy profiles as a function of , and , as an example. The peak value of the transition state region was used as the cutoff distance to define these states. Thus, 0.96 nm, 0.93 ns and 0.86 nm are used to define the I36I36, I36I44 and I44I44 substates, respectively. (PDF) [file pcbi.1003691.s009.pdf]

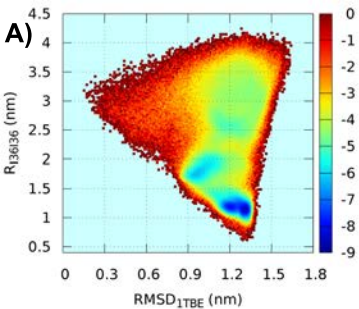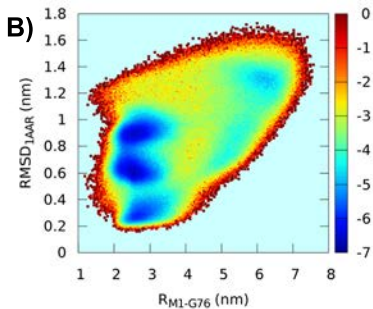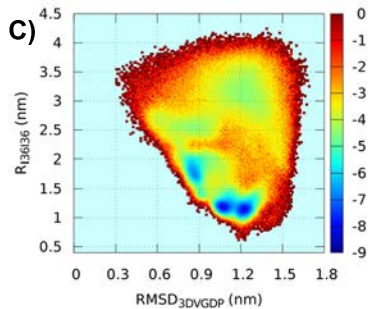

Supplement: Figure S10 — Three intermediate states revealed on the free energy surface of K48-diUb. (PDF) [file pcbi.1003691.s010.pdf]

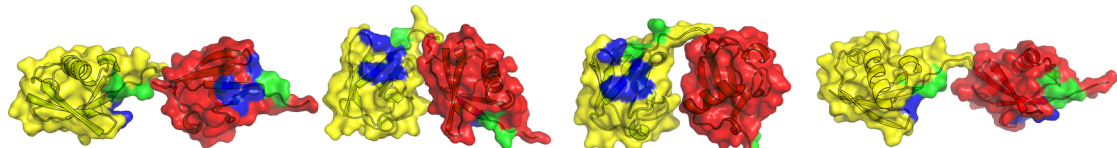

**G76-K63 (open)**

**G76-K63 (compact)**

**G76-M1 (compact)**

**G76-M1 (open)**

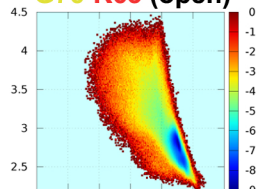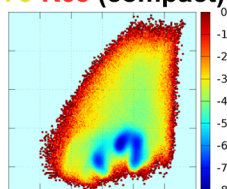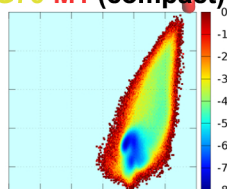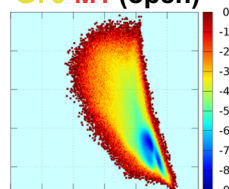

**K48**

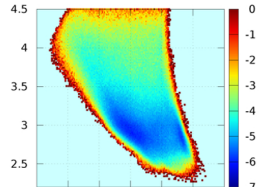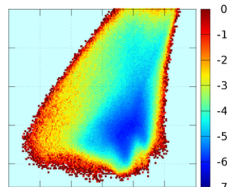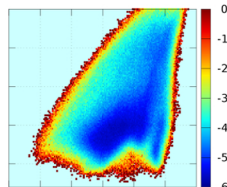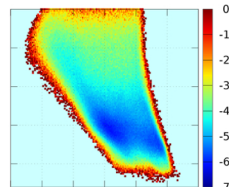

**K63**

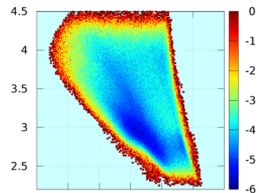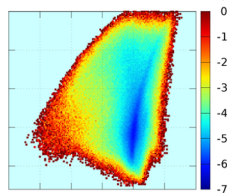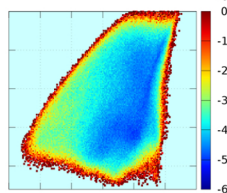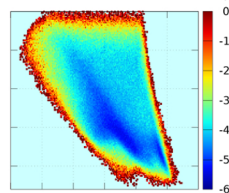

**M1**

**3H7P**

**3DVG**

**3AXC**

**2W9N**

**RMSD (nm)**

**Rcom (nm)**

Supplement: Figure S11 — The free energy surfaces between K63-diUb and linear diUb are highly similar to each other but significantly different from K48-diUb. The free energy surfaces were plotted as a function of and . X represents the X-ray structures of 3H7P, 3DVG, 3AXC and 2W9N which are shown above as the open and compact conformations of M1- and K63-diUbs. The x axis corresponds to (in unit of nm). The y axis corresponds to (in unit of nm). The free energy surfaces of K63-diUb and M1-diUb are almostly identical with each other, however significantly distinct from that of K48-diUb. Note that the corresponding linkage models were used to sample the conformational spaces of K48-, K63- and M1-linked diUbs. (PDF) [file pcbi.1003691.s011.pdf]

**A)**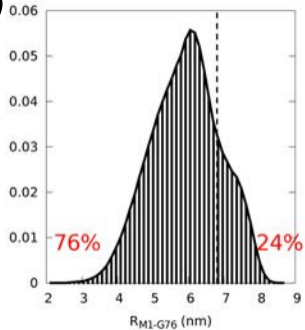**B)**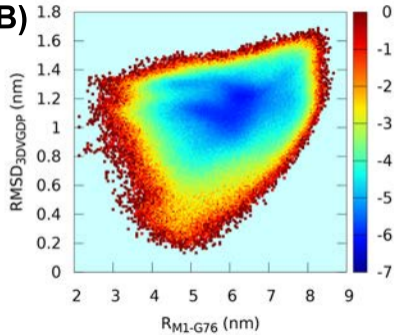**C)**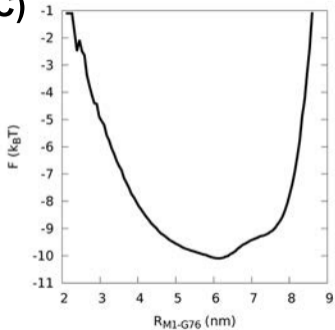

Supplement: Figure S12 — Conformational distribution of K63-diUb based on the order parameter similar to smFRET measurement. It is difficult to monitor the conformational space of K63-diUb via this order parameter. However, the populations of open and compact states are estimated to be 24% and 76%, respectively. This is quantitatively consistent with the smFRET data. Despite of this, it is better to investigate the conformational dynamics of diUb with more than dye pairs because one-dimensional conformational distribution or free energy profile have been suggested to be not sufficient to describe the multi-state landscape of many proteins. (PDF) [file pcbi.1003691.s012.pdf]

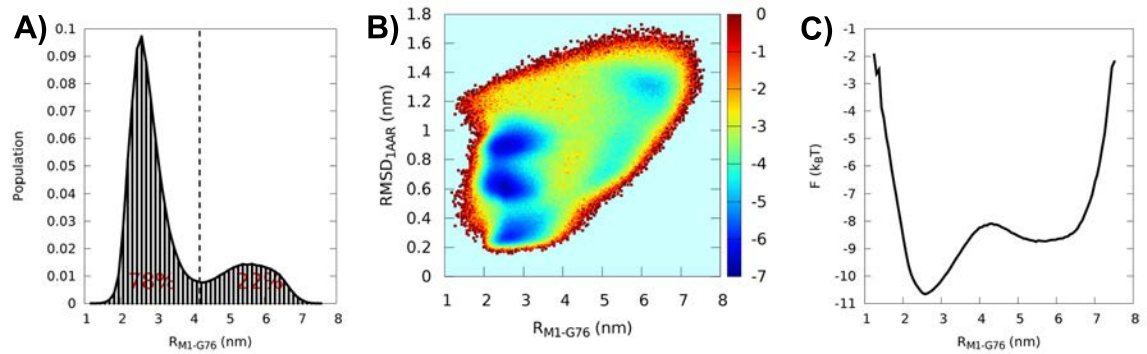

Supplement: Figure S13 — Evidence that intermediate states are possibly hidden on the one-dimensional conformational distribution or free energy profiles but can be detected by multi-dimensional free energy profiles. Note that the data is from the simulation of K48-diUb model. (PDF) [file pcbi.1003691.s013.pdf]

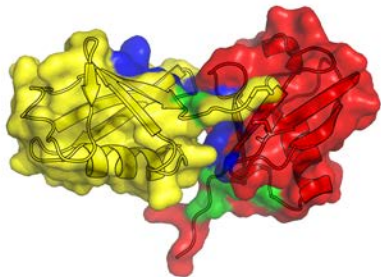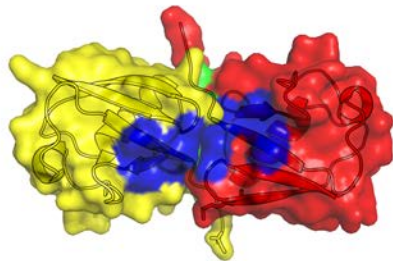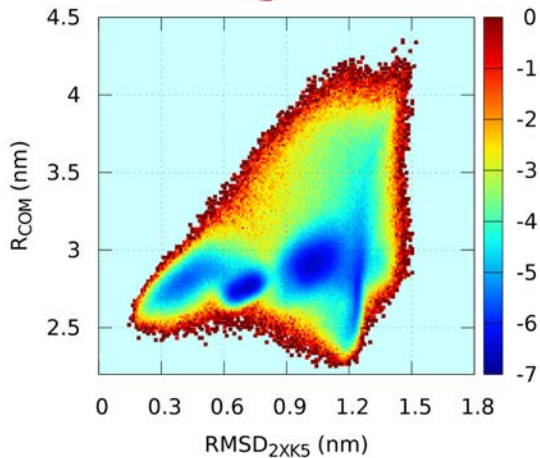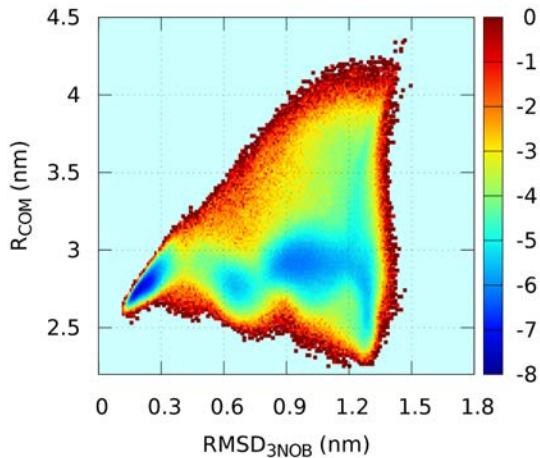

Supplement: Figure S14 — Functional landscape of K33-diUb. K33-diUb only can form the similar conformation to the compact state of K6 with I36–I44 interface (PDB 2XK5) but also is able to sample the similar conformation of the compact state of K11 with I36-I36 interface (PDB 3NOB). (PDF) [file pcbi.1003691.s014.pdf]

Autocorrelation

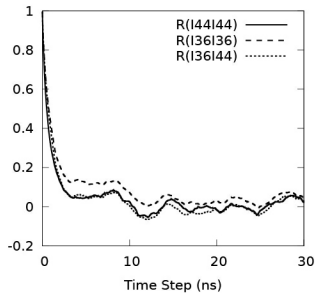

(A)

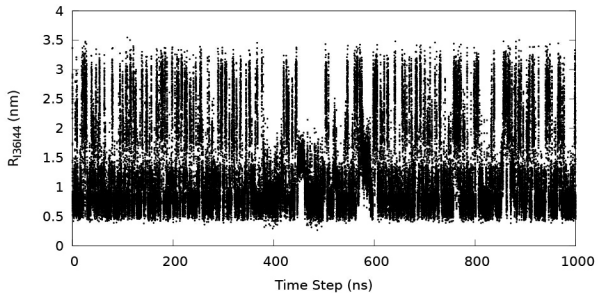

(B)

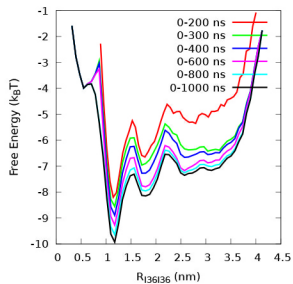

(C)

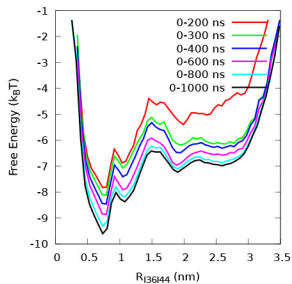

(D)

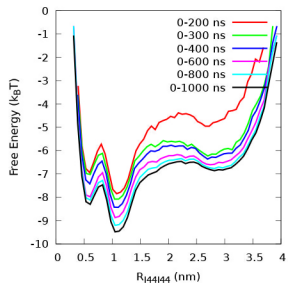

(E)

Supplement: Figure S15 — Convergence analysis of single independent simulation. As a typical simulation, one of the MD trajectories of K48-diUb model was chosen to show the result. The trajectory was projected onto three observables (, and ), (A) Autocorrelation functions of , and . (B) A typical MD trajectory of . The autocorrelation analysis indicates the correlation time of these observables is about 10 ns. Note that the correlation time measures the length of simulation time required for the trajectory to lose correlation with earlier observables. The ratio of the total simulation time to the correlation time can validate if the simulation has sufficient statistically independent observables, so as to provide an estimation of the sampling quality. Thus, in our simulation, suggests a good sampling. (C–E) Free energy profiles of , and as a function of different simulated lengths ranging from 200 ns to 1000 ns. We can see that the curve of free energy profiles is well conserved after 300 ns. This also indicates convergent results. In addition, the symmetry of interfacial interactions between Ub units also suggests the sufficient sampling of the simulations of the free Ub model. (PDF) [file pcbi.1003691.s015.pdf]

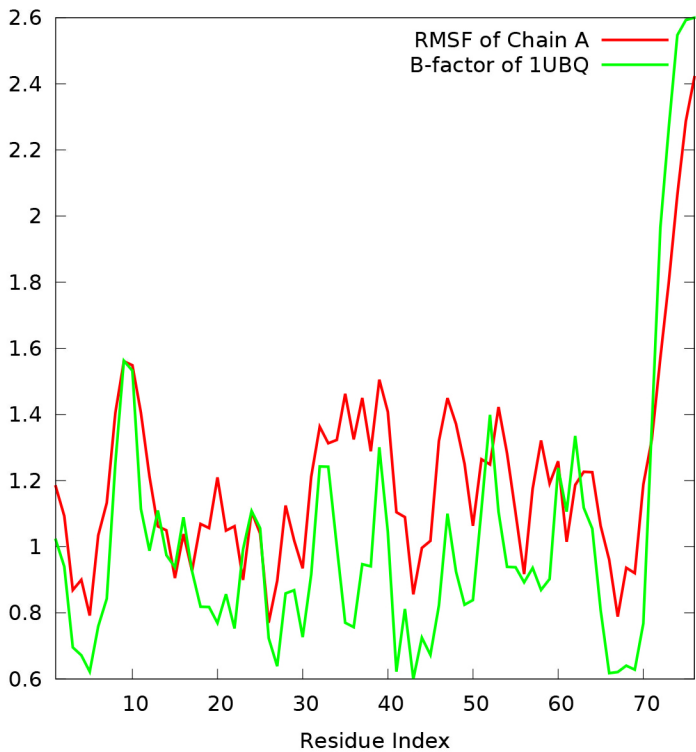

Supplement: Figure S16 — Comparison between conformational fluctuation of Ub units with experimental temperature factor. (PDF) [file pcbi.1003691.s016.pdf]
