# Supplementary material for: PolyUbiquitin Chain Linkage Topology Selects the Functions from the Underlying Binding Landscape
Source: PLoS Comput Biol. 2014 Jul 3;10(7):e1003691. doi: 10.1371/journal.pcbi.1003691 (PMC4081019; doi:10.1371/journal.pcbi.1003691)
Supplement: Figure S5 — Entropy-enthalpy compensation analysis by decomposing free energy F(x) into (x) and TS(x). Here x is . (PDF) [file pcbi.1003691.s005.pdf]

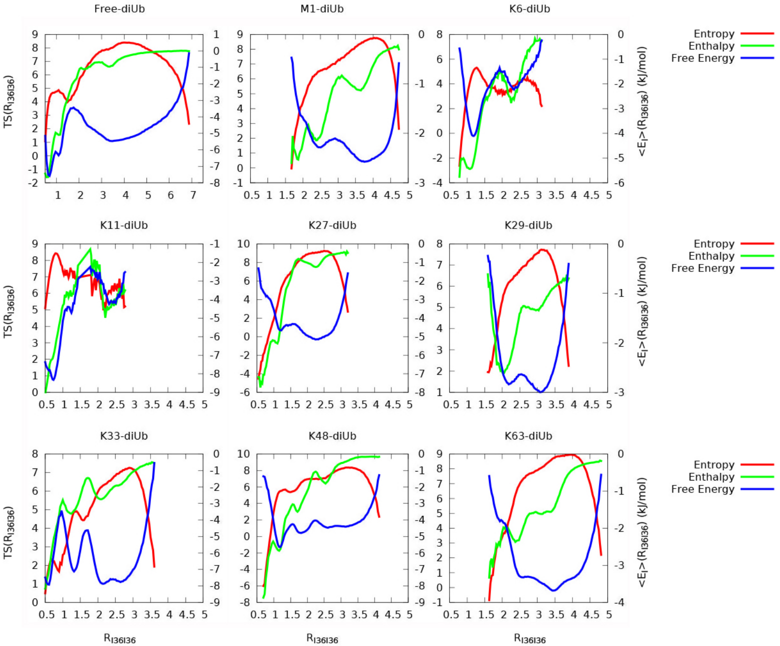
Free-diUb
M1-diUb
K6-diUb
K11-diUb
K27-diUb
K29-diUb
K33-diUb
K48-diUb
K63-diUb
Entropy
Enthalpy
Free Energy
TS(R$_{I36I36}$)
<E$_I$>(R$_{I36I36}$) (kJ/mol)
R$_{I36I36}$
