# Supplementary material for: PolyUbiquitin Chain Linkage Topology Selects the Functions from the Underlying Binding Landscape
Source: PLoS Comput Biol. 2014 Jul 3;10(7):e1003691. doi: 10.1371/journal.pcbi.1003691 (PMC4081019; doi:10.1371/journal.pcbi.1003691)
Supplement: Figure S17 — The matrix of hydrophobic parameters for different sidechain-sidechain interactions in the protein-protein association model. (PDF) [file pcbi.1003691.s017.pdf]

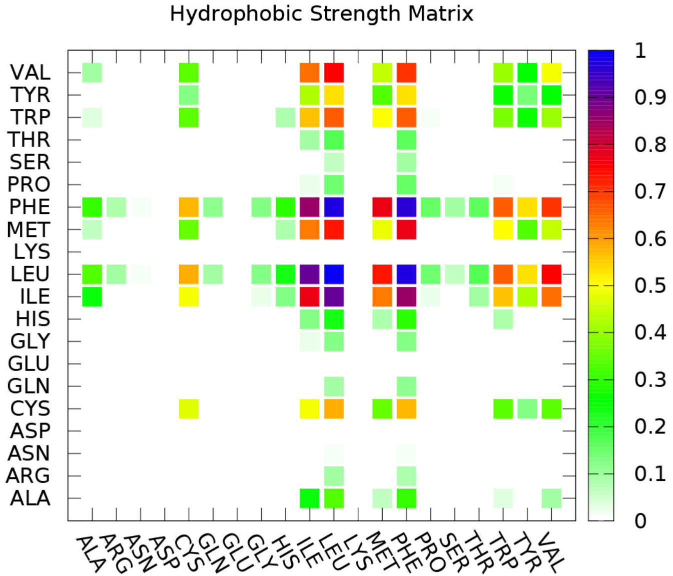

Hydrophobic Strengths for Residue Pairs

ALA	ILE	0.24	MET	ILE	0.63	ILE	HIS	0.12	THR	LEU	0.17
ALA	LEU	0.33	MET	LEU	0.73	ILE	ILE	0.77	THR	PHE	0.16
ALA	MET	0.06	MET	MET	0.48	ILE	LEU	0.9	TRP	ALA	0.03
ALA	PHE	0.3	MET	PHE	0.77	ILE	MET	0.63	TRP	CYS	0.34
ALA	TRP	0.03	MET	TRP	0.5	ILE	PHE	0.85	TRP	HIS	0.08
ALA	VAL	0.09	MET	TYR	0.33	ILE	PRO	0.02	TRP	ILE	0.56
ARG	LEU	0.09	MET	VAL	0.44	ILE	THR	0.09	TRP	LEU	0.66
ARG	PHE	0.08	PHE	ALA	0.3	ILE	TRP	0.56	TRP	MET	0.5
ASN	LEU	0.01	PHE	ARG	0.08	ILE	TYR	0.42	TRP	PHE	0.67
ASN	PHE	0.01	PHE	ASN	0.01	ILE	VAL	0.64	TRP	PRO	0.09
CYS	CYS	0.47	PHE	CYS	0.57	LEU	ALA	0.33	TRP	TRP	0.37
CYS	ILE	0.49	PHE	GLN	0.11	LEU	ARG	0.09	TRP	TYR	0.26
CYS	LEU	0.58	PHE	GLY	0.12	LEU	ASN	0.01	TRP	VAL	0.4
CYS	MET	0.35	PHE	HIS	0.29	LEU	CYS	0.58	TYR	CYS	0.12
CYS	PHE	0.57	PHE	ILE	0.85	LEU	GLN	0.09	TYR	ILE	0.42
CYS	TRP	0.34	PHE	LEU	0.97	LEU	GLY	0.12	TYR	LEU	0.53
CYS	TYR	0.12	PHE	MET	0.77	LEU	HIS	0.23	TYR	MET	0.33
CYS	VAL	0.34	PHE	PHE	0.96	LEU	ILE	0.9	TYR	PHE	0.53
GLN	LEU	0.09	PHE	PRO	0.15	LEU	LEU	0.99	TYR	TRP	0.26
GLN	PHE	0.11	PHE	SER	0.09	LEU	MET	0.73	TYR	TYR	0.13
GLY	ILE	0.02	PHE	THR	0.16	LEU	PHE	0.97	TYR	VAL	0.25
GLY	LEU	0.12	PHE	TRP	0.67	LEU	PRO	0.14	VAL	ALA	0.09
GLY	PHE	0.12	PHE	TYR	0.53	LEU	SER	0.06	VAL	CYS	0.34
HIS	ILE	0.12	PHE	VAL	0.7	LEU	THR	0.17	VAL	ILE	0.64
HIS	LEU	0.23	PRO	ILE	0.02	LEU	TRP	0.66	VAL	LEU	0.75
HIS	MET	0.08	PRO	LEU	0.14	LEU	TYR	0.53	VAL	MET	0.44
HIS	PHE	0.29	PRO	PHE	0.15	LEU	VAL	0.75	VAL	PHE	0.7
HIS	TRP	0.08	PRO	TRP	0.01	MET	ALA	0.07	VAL	TRP	0.4
ILE	ALA	0.24	SER	LEU	0.06	MET	CYS	0.35	VAL	TYR	0.25
ILE	CYS	0.49	SER	PHE	0.09	MET	HIS	0.08	VAL	VAL	0.49
ILE	GLY	0.02	THR	ILE	0.09						
