# Supplementary material for: PolyUbiquitin Chain Linkage Topology Selects the Functions from the Underlying Binding Landscape
Source: PLoS Comput Biol. 2014 Jul 3;10(7):e1003691. doi: 10.1371/journal.pcbi.1003691 (PMC4081019; doi:10.1371/journal.pcbi.1003691)
Supplement: Text S1 — Supporting Methods (Estimation of disassociation constant, Entropy reduction estimation by a polymer theory); Tables 14; Supporting References. (PDF) [file pcbi.1003691.s018.pdf]

Text S1: Polyubiquitin Chain Linkage Topology Selects the Functions From the Underlying Binding Landscape

Yong Wang [*], **Chun Tang** [†], **Erkang Wang** [*], **and Jin Wang** [*‡§]

[*]State Key Laboratory of Electroanalytical Chemistry, Changchun Institute of Applied Chemistry, Chinese Academy of Sciences, Changchun, Jilin, 130022, P.R. China,[†]CAS Key Laboratory of Magnetic Resonance in Biological Systems, State Key Laboratory of Magnetic Resonance and Atomic and Molecular Physics, Wuhan Institute of Physics and Mathematics, Chinese Academy of Sciences, Wuhan, Hubei 430071, China,[‡]College of Physics, Jilin University, Changchun, Jilin, P.R. China, and [§]Department of Chemistry and Physics, State University of New York at Stony Brook, Stony Brook, New York 11794-3400, USA

Submitted to PLOS Computational Biology

Estimation of disassociation constant. To estimate the equilibrium disassociation constant K_D reflecting binding affinity between Ub monomers, we consider two-state kinetics for the formation of the Ub dimer at equilibrium, which may be expressed as,

$$[U] + [U] \underset{k_{off}}{\overset{k_{on}}{\rightleftharpoons}} [D]$$

where k_{on} is the second-order rate constant as the association rate, while k_{off} is the first order rate constant for the dissociation. The ratio between the two rate constants yields the equilibrium disassociation constant k_D (has units of concentration) given by:

$$\begin{aligned} k_D &= \frac{k_{off}}{k_{on}} = \frac{[U]*[U]}{[D]} \\ &= \frac{P_f^2}{0.5(1-P_f)} * [U_0] \\ &= \frac{2P_f^2}{1-P_f} * \frac{1}{V} \\ &\approx \frac{2P_f^2}{1-P_f}\frac{400}{R^3}[M] \end{aligned}$$

We define the concentration of free Ub as $[U_f]$, so total Ub concentration $[U_0] = [U_f] + 2[D]$. $P_f = \frac{[U_f]}{[U_0]}$ is the population of free Ub, then $[D] = 0.5(1 - P_f)$. R in unit of $\mathring{A}$ is radius of the effective simulation spherical box. $\frac{400}{R^3}$ in the equation is a result of unit conversion from $1 molecule/\mathring{A}^3$ to $1 mol/L$. After the parameter calibration, the binding affinity was estimated to be between 0.6 and 1.9 mM which is about 2.5 times stronger than experimental measurement. Considering that the estimation of $[U_0]$ does not take into account the excluded volume of Ubs themselves, the accurate value of k_D should be slightly higher than the current value. In total, the binding affinity in simulation is reasonable consistent with experimental measurement.

Entropy reduction estimation by a polymer theory. The free energy contribution of enthalpy term resulted from bond constraint can be estimated to be about $2.2k_BT$ given that the energy parameter in our model for a typical contact is 0.92KJ/mol and the simulation temperature is 50K. Then we applied a polymer theory (*1*) to estimate the free energy contribution of entropy reduction resulted from the introduction of an iso-peptide bond between two Ub units by the expression: $-ln(p(u))$ (in unit of k_BT). Here, $p(u) = (\frac{3\mu}{4\pi N})^{\frac{3}{2}}\frac{4\pi}{3}(\frac{I}{b})^3$ is the average probability to form a bond/contact constraint with μ contacts/bonds already present, where N is the residue number, b is the persistent length of a peptide chain, and I is the length of the bonded constraint. For our case, N=152 and I=4.0$\mathring{A}$.

Figure S4 shows $p(\mu, b)$ as a function of μ and b. μ was varied from 1 to 59. Note that 59 is the number of interfacial contacts in closed diUb (PDB 1AAR) representing the upper limitation. b was varied from from 6 to 14 $\mathring{A}$, considering that a typical b for polyethylene is 14 and a typical b for Ub is 12 (*2*). The plot leads to a robust observation that the entropic contribution is larger than the enthalpic contribution. In contrast to the free model, the only difference of the linkage models is an extra bond constraint. In total, the quantified analysis based on polymer theory supports that the impact of the bonded constraint on the free energy landscape of diUbs is mainly entropic rather than enthalpic.

To further quantify the impact of bonded constraints on different linkage types, we performed a detailed analysis of entropy-enthalpy compensation of all 9 models. We calculated the entropic term TS(x) by $< E_I > (x) - F(x)$. Note that $< E_I > (x)$ and F(x) are the interfacial energy profile and the free energy profile as a function of the binding reaction coordinate x, respectively. To check the robustness, we used three order parameters R_{COM}, R_{I36I36}, and R_{I44I44} as the binding reaction coordinates. The results are shown in Fig. S5-7. To quantify the contribution of entropy and enthalpy to the free energy landscape, we performed correlation coefficient analysis, as shown in Table S5.

1. Plotkin, S. S, Wang, J, & Wolynes, P. G. (1996) Correlated energy landscape model for finite, random heteropolymers Phys Rev E 53, 6271.
2. Gra ter, F, Heider, P, Zangi, R, & Berne, B. (2008) Dissecting entropic coiling and poor solvent effects in protein collapse J Am Chem Soc 130, 11578–11579.

Reserved for Publication Footnotes

Table S1: Experimental Structures of diUbs

Linkage Type	PDB	Structure	Reference
M1	2W9N	open	Komander2009
	3AXC	compact	Rohain2012
K6	2XK5, 3ZlZ [a]	compact [e]	Virdee2010, Hospenthal2013
K11	3NOB [b], 2XEW [c]	compact [e]	Matsumoto2010, Bremm2010
K27	No available structure		
K29	No available structure		
K33	No available structure		
K48	1F9J	open	Philips2001
	2PE9	compact [f]	Ryabov2007
	1TBE, 3AUL, 3NS8	compact	Cook1994, Hirano2011, Lai2012
	1AAR, 2PEA, 2BGF	closed [g]	Cook1992, Ryabov2007, DijK2005
K63	3H7P, 2JF5[d]	open	Weeks2009, Komander2009
	3DVG	compact	Newton2008

[a] A mirror structure of 2XK5.
[b] A and B chains of 3NOB.
[c] J and K chains of 2XEW, similar to 3NOB.
[d] A mirror structure of 3H7P.
[e] Interface formed between I36 patch of distal Ub and I36 patch of proximal Ub.
[f] Interface formed between I36 patch of distal Ub and I44 patch of proximal Ub.
[g] Interface formed between I44 patch of distal Ub and I44 patch of proximal Ub.

Table S2: Theoretical Models for Different Ub Systems

Theoretical Model	Description [a]
Free	No linkage between Ub units which are modeled by SBM
CGK6	add an isopeptide bond between G76 and K6
CGK11	add an isopeptide bond between G76 and K11
CGK27	add an isopeptide bond between G76 and K27
CGK29	add an isopeptide bond between G76 and K29
CGK33	add an isopeptide bond between G76 and K33
CGK48	add an isopeptide bond between G76 and K48
CGK63	add an isopeptide bond between G76 and K63
CGM1	add a peptide bond between G76 of distal Ub and M1 of proximal Ub, and additional bond angle and dihedral angle terms

[a] an isopeptide bond is formed between a carboxyl group of Ub G76 (distal Ub) and an ϵ-amino group of another Ub's lysine (proximal Ub).

Table S3: Pre-existence of X-ray or NMR structures of diUbs on the binding landscape of free Ub monomers

Model	X-ray or NMR structure	Linkage	Minimal RMSD[a]	State
Free	3AXC	M1	0.25	compact
	2XK5	K6	0.17	compact, I44-I36
	2XK5-Mirror [b]	K6	0.21	compact, I36-I44
	2XEW or 3NOB [c]	K11	0.15	compact, I36-I36
	1AAR or 2PEA [d]	K48	0.26	closed, I44-I44
	1TBE [e]	K48	0.35	compact
	3AUL [e]	K48	0.35	compact
	3NS8 [e]	K48	0.34	compact
	2W9N	M1	0.16	open
	3DVG	K63	0.32	open
	3H7P	K63	0.21	open

[a] in unit of nm.
[b] The mirror structure of 2XK5 was modeled from 3ZLZ which has an interface formed between I36 patch of distal Ub and I44 patch of proximal Ub.
[c] Conformation of 3NOB (A and B chains) is similar to that of 2XEW (J and K chains).
[d] We only measured the RMSD to 1AAR due to the similarity between the conformation of 2PEA and that of 1AAR.
[e] Conformations of 1TBE, 3AUL and 3NS8 are similar.

Table S4: Structure prediction by different linkage models

Model	X-ray or NMR structure	Minimal RMSD	Linkage	State
CGK48	1AAR, 2PEA	0.16	K48	closed, I44-I44
	1F9J	0.18	K48	open
	1TBE	0.25	K48	compact
	3AUL	0.25	K48	compact
	3NS8	0.25	K48	compact
	2XK5	0.10	K6	compact, I36-I44
	3NOB	0.67	K11	compact, I36-I36
CGK63	3H7P	0.15	K63	open
	2JF5	0.13	K63	open
	3DVG	0.14	K63	compact
	3AXC	0.21	M1 [a]	compact
	2W9N	0.12	M1 [a]	open
CGM1	3AXC	0.14	M1	compact
	2W9N	0.11	M1	open
	3H7P	0.14	K63	open
	2JF5	0.15	K63	open
	3DVG	0.25	K63	compact
CGK11	3NOB or 2XEW	0.11	K11	compact, I36-I36
	2XK5	0.29	K6	compact, I44-I36
CGK6	2XK5	0.29	K6	compact, I44-I36
	3NOB	0.18	K11	compact, I36-I36
	1AAR	0.26	K48	closed, I44-I44
CGK27	No Available Structure of K27-diUb			
	2XK5	0.44	K6	compact, I44-I36
	3NOB	0.49	K11	compact, I36-I36
	1AAR	0.25	K48	closed, I44-I44
CGK29	No Available Structure of K29-diUb			
	2XK5	0.39	K6	compact, I44-I36
	3NOB	0.47	K11	compact, I36-I36
	1AAR	0.84	K48	closed, I44-I44
CGK33	No Available Structure of K33-diUb			
	2XK5	0.15 [b]	K6	compact, I44-I36
	3NOB	0.12 [b]	K11	compact, I36-I36
	1AAR	0.73	K48	closed, I44-I44

[a] K63- and linear diUb can sample the similar conformation space.

[b] We predicted that K33-diUb can form the conformations similar to the compact structures of K6- and K11-diUb.

Table S5. Correlation Coefficient Analysis

Correlation Coefficient between Free Energy and Entropy (CC_{FS})			
Model	R_{COM}	R_{I44I44}	R_{I36I36}
Free	0.29	0.18	0.37
M1	0.91	0.95	0.94
K6	0.42	0.52	0.49
K11	0.49	0.44	0.27
K27	0.85	0.77	0.87
K29	0.92	0.89	0.94
K33	0.58	0.58	0.51
K48	0.51	0.37	0.67
K63	0.88	0.95	0.94

Correlation Coefficient between Free Energy and Enthalpy (CC_{FE})			
Model	R_{COM}	R_{I44I44}	R_{I36I36}
Free	0.41	0.44	0.35
M1	0.08	0.29	0.35
K6	0.89	0.89	0.72
K11	0.74	0.85	0.68
K27	0.06	0.06	0.58
K29	0.26	0.09	0.30
K33	0.51	0.03	0.09
K48	0.53	0.56	0.06
K63	0.18	0.07	0.22
